# Targeted theranostic photoactivation on atherosclerosis

**DOI:** 10.1186/s12951-021-01084-z

**Published:** 2021-10-24

**Authors:** Joon Woo Song, Jae Won Ahn, Min Woo Lee, Hyun Jung Kim, Dong Oh Kang, Ryeong Hyun Kim, Un Gyo Kang, Yeon Hoon Kim, Jeongmoo Han, Ye Hee Park, Hyeong Soo Nam, Hongki Yoo, Kyeongsoon Park, Jin Won Kim

**Affiliations:** 1 Multimodal Imaging and Theranostic Laboratory, Cardiovascular Center, Korea University Guro Hospital, 148 Gurodong-ro, Guro-gu, Seoul, 08308 Republic of Korea; 2 Department of Systems Biotechnology, Chung-Ang University, Anseong, Gyeonggi-do 17546 Republic of Korea; 3 Department of Biomedical Engineering, Hanyang University, 222 Wangsimni-ro, Seongdong-gu, Seoul, 04763 Republic of Korea; 4 Department of Mechanical Engineering, orea Advanced Institute of Science and Technology, 291 Daehak-ro, Yuseong-gu, Daejeon, 34141 Republic of Korea

**Keywords:** Macrophage targetable phototheranostic agent, Photodynamic therapy, Foam cells, Atherosclerosis, Autophagy, Efferocytosis

## Abstract

**Background:**

Photoactivation targeting macrophages has emerged as a therapeutic strategy for atherosclerosis, but limited targetable ability of photosensitizers to the lesions hinders its applications. Moreover, the molecular mechanistic insight to its phototherapeutic effects on atheroma is still lacking. Herein, we developed a macrophage targetable near-infrared fluorescence (NIRF) emitting phototheranostic agent by conjugating dextran sulfate (DS) to chlorin e6 (Ce6) and estimated its phototherapeutic feasibility in murine atheroma. Also, the phototherapeutic mechanisms of DS-Ce6 on atherosclerosis were investigated.

**Results:**

The phototheranostic agent DS-Ce6 efficiently internalized into the activated macrophages and foam cells via scavenger receptor-A (SR-A) mediated endocytosis. Customized serial optical imaging-guided photoactivation of DS-Ce6 by light illumination reduced both atheroma burden and inflammation in murine models. Immuno-fluorescence and -histochemical analyses revealed that the photoactivation of DS-Ce6 produced a prominent increase in macrophage-associated apoptotic bodies 1 week after laser irradiation and induced autophagy with Mer tyrosine-protein kinase expression as early as day 1, indicative of an enhanced efferocytosis in atheroma.

**Conclusion:**

Imaging-guided DS-Ce6 photoactivation was able to in vivo detect inflammatory activity in atheroma as well as to simultaneously reduce both plaque burden and inflammation by harmonic contribution of apoptosis, autophagy, and lesional efferocytosis. These results suggest that macrophage targetable phototheranostic nanoagents will be a promising theranostic strategy for high-risk atheroma.

**Graphical abstract:**

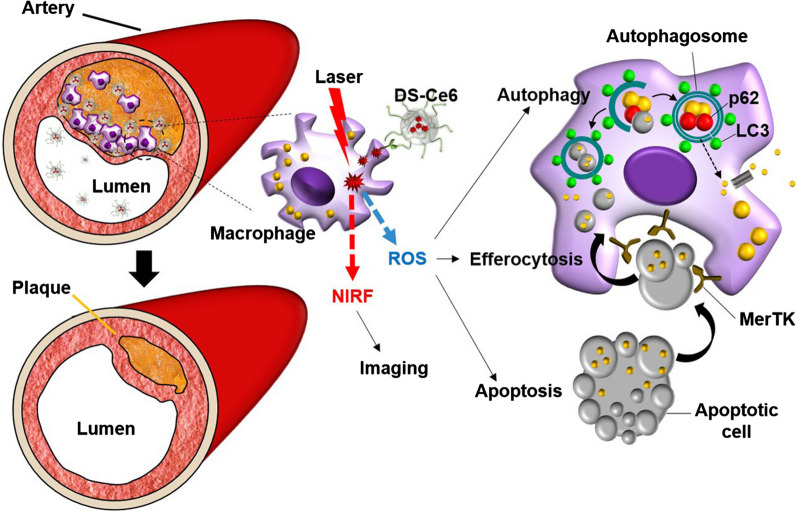

**Supplementary Information:**

The online version contains supplementary material available at 10.1186/s12951-021-01084-z.

## Background

Atherosclerotic cardiovascular disease (ASCVD) is the leading cause of death worldwide [1]. While lipid lowering strategies facilitating a stabilization of high-risk atheroma have been developed, the residual risk of ASCVD is still substantial [2]. Moreover, the accurate detection of inflamed plaque has proven challenging, whereas multimodal catheter approach was able to image the inflammatory activities in the coronary beds [3].

Macrophages play crucial roles in the development of atherosclerosis, from initiation to fatal thrombotic rupture [4, 5]. In the artery, the differentiated macrophages from monocytes take up the lipid. Then, these macrophage-derived foam cells are presented at initial stages in the development of atherosclerosis [6, 7]. Photoactivation, the process of activating a photosensitizer by means of laser irradiation, has emerged as a promising therapeutic strategy for atherosclerosis therapy because this approach reduces activated macrophages in atheroma and thus stabilizes atherosclerotic plaques [8–10]. Photoactivation utilizes a specific wavelength of light to activate photosensitizers, converting oxygen to reactive oxygen species (ROS) such as singlet oxygen (^1^O_2_) [8]. ROS generated by light energy eradicated the inflammatory cells via induction of apoptosis, and even promoted the vascular healing [9, 11, 12]. While the biological effects of photoactivation on atheroma are compelling, indeed, the use of currently available photosensitizers is still challenging. The non-specific binding of the photosentizing agent to the vascular structures could potentially cause unexpected damage to the protective barriers, including smooth muscle cells (SMCs) [13, 14] and endothelial cells (ECs) [14, 15]. Damaged SMCs and endothelial barrier impair the integrity of the fibrous cap overlying the atheroma leading to thrombosis and rupture [16, 17]. Therefore, a novel photoactivatable agent with a specific targeting affinity to macrophage needs to be developed.

Another issue of photoactivation effects on atherosclerosis is the ultimate fate of ROS-induced apoptotic materials. While the mechanism of the anti-atherogenic action of photoactivation is unclear, macrophage apoptosis is considered as the main cellular process for the diminished inflammation to stabilize the high-risk atheroma [9, 18–20]. However, the phagocytic clearance of apoptotic cells by macrophages, known as efferocytosis, is reported to be defective in advanced atheroma and accordingly, rather, suppression of apoptosis might be associated with less plaque burden [21, 22]. Thus, it remains uncertain whether photoactivation-mediated inflammatory cell death is indeed beneficial, resulting in stabilization of the inflamed atheroma. Of particular interest is autophagy activation produced by light energy, which promotes the degradation of cytoplasmic components in lysosomes [23, 24]. Reportedly, ROSs generated by photoactivation induce autophagy in foam cells [25]. Considering that the autophagy machinery is crucial in the degradation of phagocytosed dying cells and defective autophagy worsens efferocytosis [26–28], hypothetically, the harmonic contribution of apoptosis, autophagy, and lesional efferocytosis induced by photoactivation could orchestrate targeted therapeutic effects on the atheroma.

**Scheme 1.**
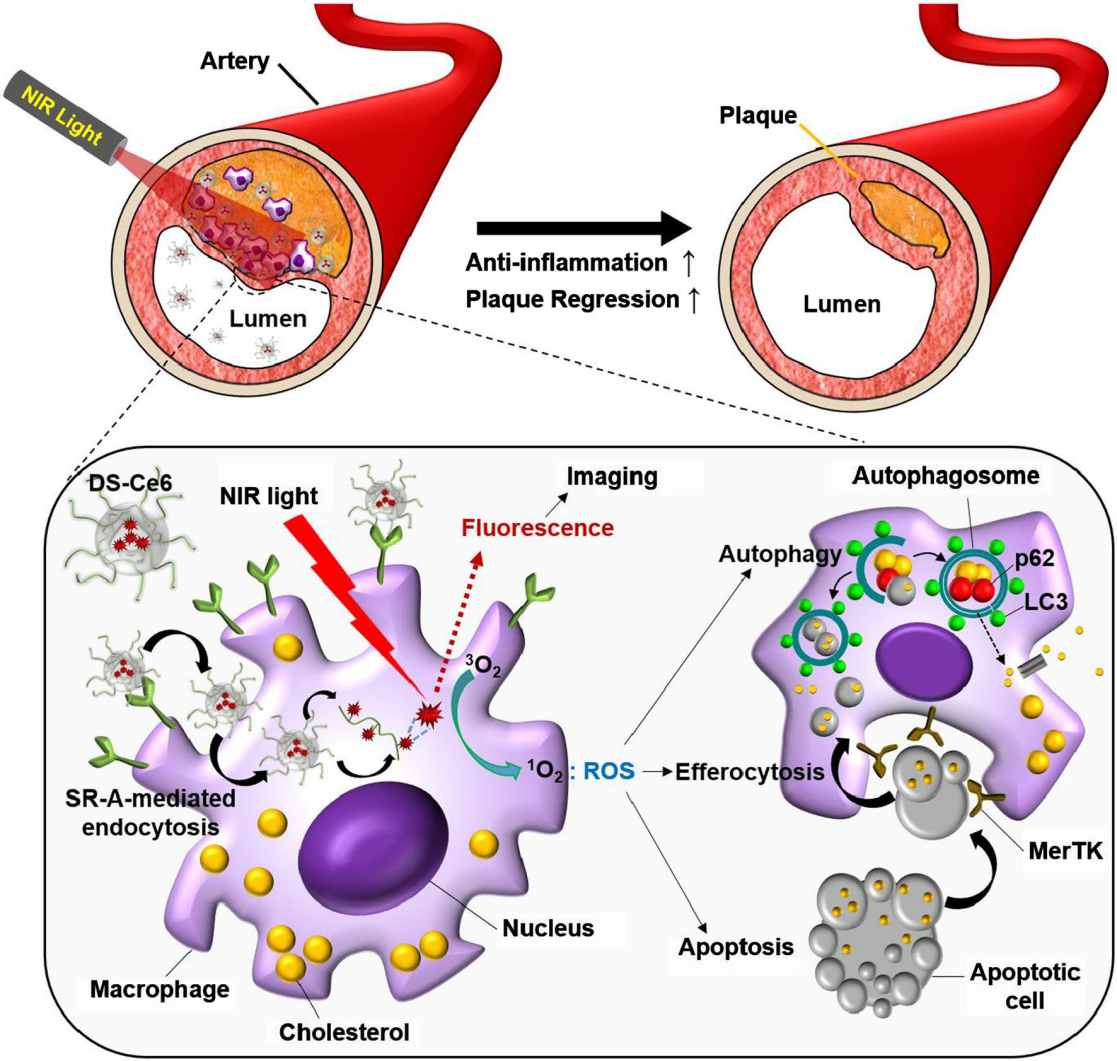
Schematic overview of the macrophage SR-A targeted photoactivation for autophagy induction and efferocytosis enhancement to regress atherosclerosis. Macrophage-targetable phototheranostic agent DS-Ce6 internalizes into the plaque macrophages through SR-A-mediated endocytosis. Upon laser irradiation, DS-Ce6 emits NIRF and simultaneously produces ROS. The generated ROS activates autophagy and upregulates MerTK expression within foam cells, and then promotes the engulfment of photoactivation-induced apoptotic cells. Macrophage-targeted photoactivation reduces inflammatory activity and results in plaque regression

Recently, protease-mediated theranostic agents [20, 29], platelet-mimicking therapeutic system [30] and macrophage targetable chitosan-based carbon nanocages [31] have been developed for photodynamic and/or photothermal therapy of atheroma, and these thernostic agents effectively alleviate the progression of atherosclerotic plaques. Except for apoptosis induction and reduction of plaque burden, these studies did not show in vivo phototherapeutic effects regarding autophagy and efferocytosis. In this study, we newly developed a theranostic photoactivation strategy for the atherosclerosis (Scheme 1). We engineered a near-infrared fluorescence (NIRF) emitting targeted photoactivatable agent (DS-Ce6) that involves dextran sulfate (DS) conjugated with the photosensitizer chlorin e6 (Ce6). DS is highly biocompatible and biodegradable with a pronounced selectivity to scavenger receptor A (SR-A) [32], a key receptor in macrophages involved in uptake of modified low-density lipoprotein (LDL) and foam cell formation [33]. A customized intravital imaging and corroborative immunostainings demonstrated the accumulation of DS-Ce6 within the murine atheroma by targeting macrophage SR-A. By in vivo serial optical imaging and comprehensive in vitro analyses, we investigated whether our targeted photoactivation strategy induces autophagy and enhances efferocytosis in light irradiated atheroma, and ultimately could reduce both plaque burden and inflammation.

## Results

### Synthesis and characteristics of SR-A targetable DS-Ce6

**Fig. 1:**
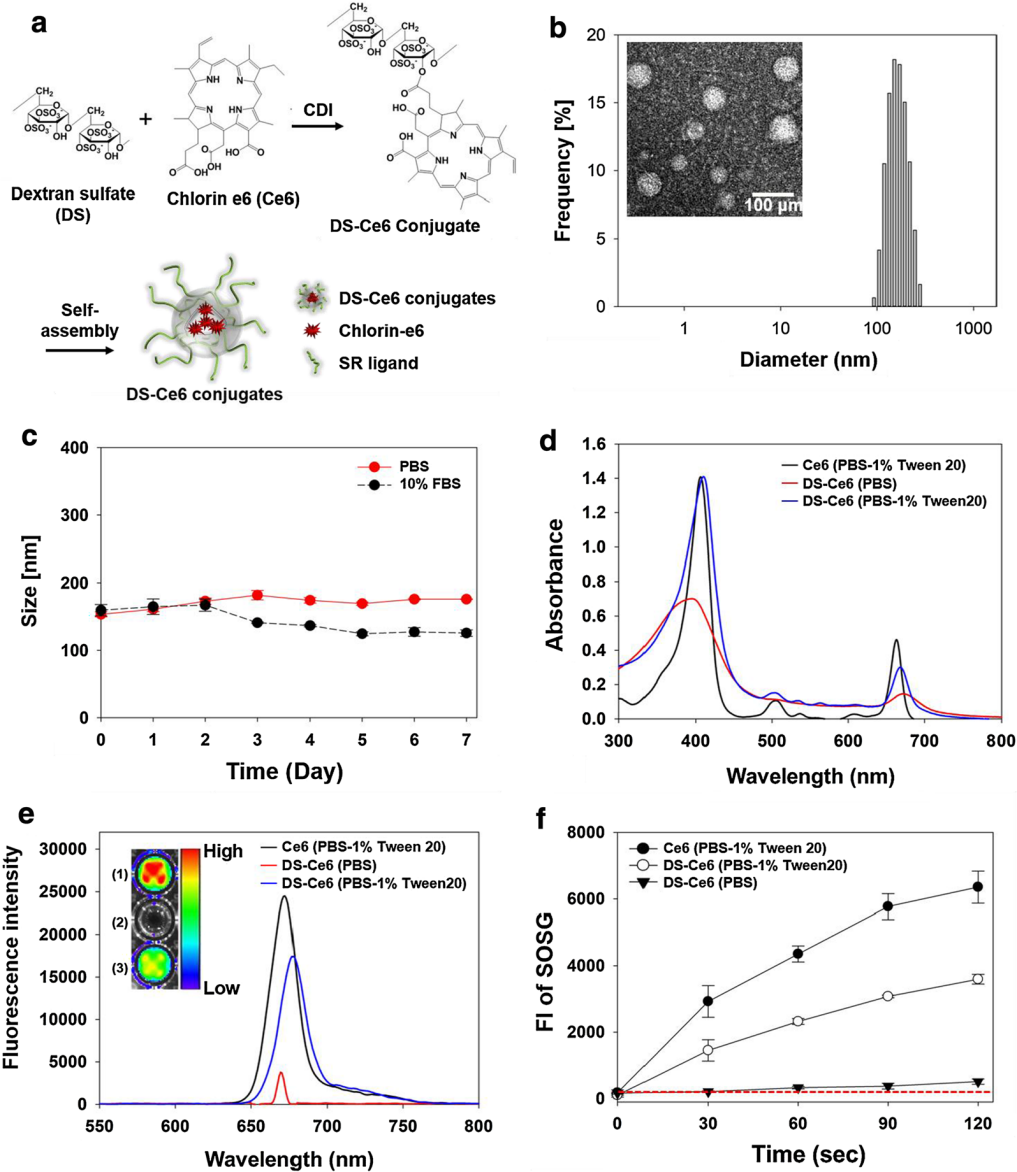
Synthesis and characteristics of macrophage SR-A-targetable DS-Ce6. **a** Chemical structure and synthetic procedure of DS-Ce6. Amphiphilic DS-Ce6 formed self-assembled nanostructure in aqueous environment. **b** Size distribution of DS-Ce6 in aqueous condition. Inset: TEM image of DS-Ce6. Scale bar = 100 nm. **c** Stability test of DS-Ce6 in PBS and 10% FBS as a function of time. **d**–**e** UV/Vis absorption **d** and fluorescence spectra **e** of Ce6 (PBS with 1% Tween 20) and DS-Ce6 (PBS only or PBS with 1% Tween 20). Inset: fluorescence images of (1) free Ce6 (PBS with 1% Tween 20), (2) DS-Ce6 (PBS) and (3) DS-Ce6 (PBS with 1% Tween 20). **f** Fluorescence intensity of SOSG of Ce6 (PBS with 1% Tween 20) and DS-Ce6 (PBS only or PBS with 1% Tween 20) as a function of the laser irradiation time

We constructed DS-Ce6 by conjugating the DS, a SR-A ligand, with the photosensitizer Ce6 using carbonyldiimidazole (CDI) chemistry. The amphiphilic DS-Ce6 forms self-assembled nanostructures in an aqueous environment, resulting in a hydrophilic outer shell organized with DS and hydrophobic inner cores with Ce6 (Fig. 1a). The number of conjugated Ce6 molecules to one mole of DS was 1.32 mol, and the calculated molecular weight of DS-Ce6 was approximately 40,789 Da. DS-Ce6 showed a characteristic peak of the C = C stretching vibration at 1593 cm^−1^, but DS did not, indicating the successful conjugation of DS with Ce6 (Additional file 1: Fig. S1). The hydrodynamic diameter of the DS-Ce6 in aqueous solution was 157.6 ± 37.8 nm (Fig. 1b). Transmission electron microscopy (TEM) images revealed that DS-Ce6 had a spherical shape with an average size of 50.1 ± 1.1 nm in dried condition (Fig. 1b, inset). Typically, the determined sizes in dried condition were much smaller than those in aqueous condition due to the shrinkage of hydrophilic shell. Meanwhile, there were no remarkable changes in the hydrodynamic mean sizes of DS-Ce6 in PBS and 10% fetal bovine serum (FBS) (Fig. 1c), indicating that DS-Ce6 is stable under biological conditions without unwanted aggregation.

The ultraviolet/visible (UV/Vis) absorption spectrum of solubilized free Ce6 in PBS containing 1% Tween 20 exhibited a sharp Soret band at 405 nm and a Q-band between 500 and 700 nm. Dispersed DS-Ce6 in PBS alone showed decreased and broadened absorption peaks owing to the interference of light transmittance by the DS-Ce6 nanostructures (Fig. 1d). Consistently, the fluorescence spectra and images revealed that DS-Ce6 in PBS alone had lower fluorescence intensity than solubilized free Ce6 because of dye-dye self-quenching due to the close proximity between Ce6 molecules within the self-assembled DS-Ce6 (Fig. 1e). However, as reported in the previous studies [34, 35], the DS-Ce6 in PBS containing 1% Tween 20 (surfactant) as the cytosol mimic environment exhibited a sharp Soret- and Q-band as well as strong fluorescence intensities because the surfactant could disrupt the dense nanostructures of DS-Ce6 (Fig. 1d, e). Meanwhile, ^1^O_2_ generation of free Ce6 in the solubilized state increased rapidly upon laser irradiation, while marginal increase was detected in PBS-only DS-Ce6. Compared to DS-Ce6 in PBS alone, DS-Ce6 in PBS containing 1% Tween 20 remarkably increased ^1^O_2_ generation. These results suggest that DS-Ce6 has a low phototoxicity in the quenched state, whereas the dequenched DS-Ce6 in the cytosol mimic environment has a high phototoxicity (Fig. 1f).

### Specific binding affinity of DS-Ce6 to SR-A-expressing macrophages

**Fig. 2:**
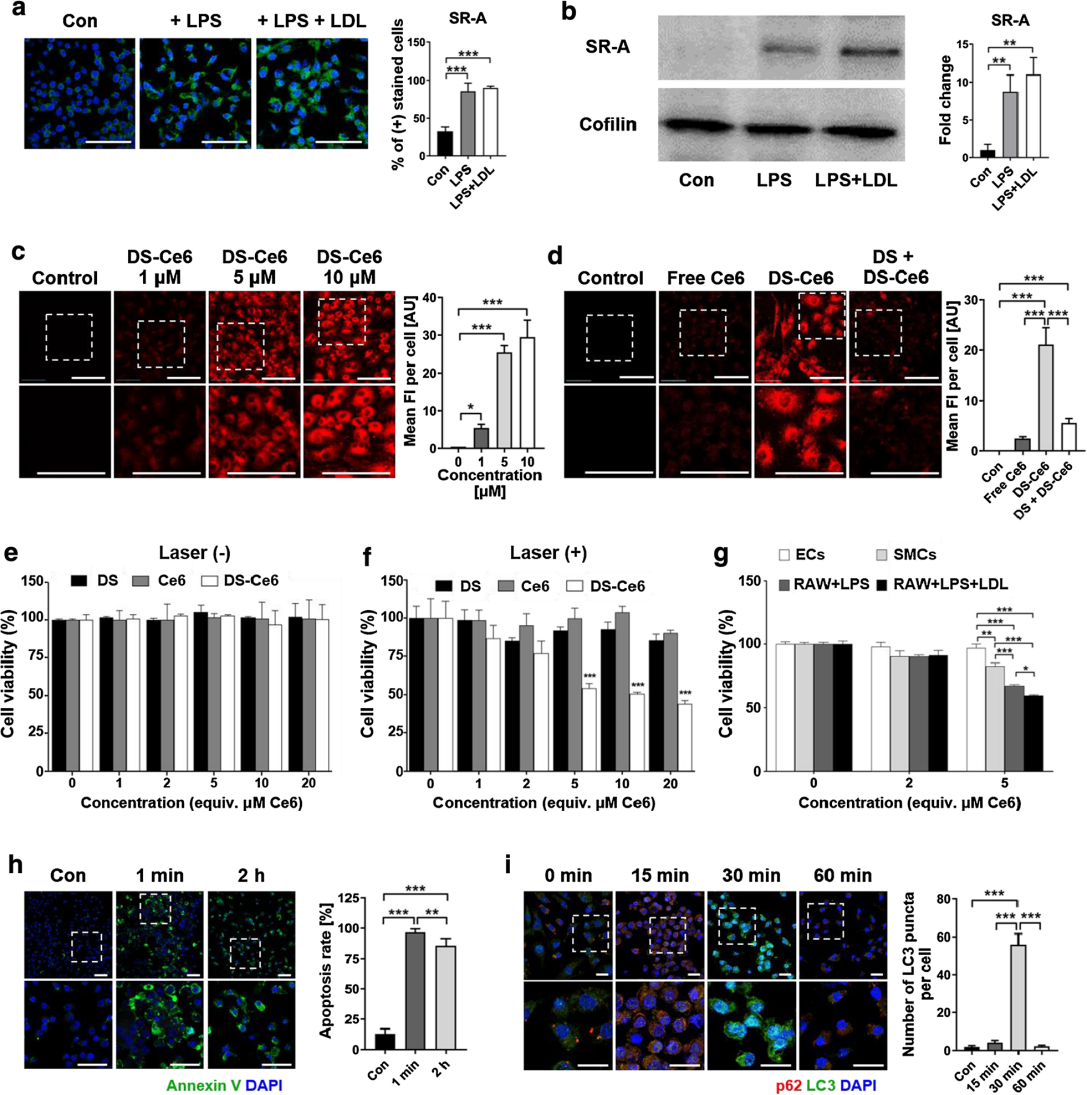
Cellular uptake of DS-Ce6 and induction of apoptotic cell death and autophagy flux by photoactivation. **a** Immunofluorescence staining of SR-A on macrophages, LPS-activated macrophages, and foam cells. The activated macrophages and foam cells were strongly positive for SR-A (green) compared to the control cells. Blue: nucleus stained with DAPI. Scale bar = 50 μm. **b** Western blot analysis of the protein expression of SR-A in control macrophages, activated macrophages, and foam cells. Quantification of SR-A normalized to cofilin, presented as fold change over controls. **c** Dose-dependent cellular uptake of DS-Ce6 in the foam cells. Scale bar = 30 μm. ^*^P < 0.05, ^***^P < 0.001. **d** Comparison of the intracellular uptake of free Ce6 (5 μM) and DS-Ce6 (equiv. 5 μM Ce6) in foam cells. To evaluate receptor-mediated endocytosis, the SR-A ligand DS was pre-treated for 1 h before DS-Ce6 incubation (DS + DS-Ce6). Scale bar = 50 μm. **e**, **f** Cell viability of the foam cells treated with different concentrations of DS-Ce6, DS, and Ce6 without **e** and with laser irradiation **f** (670 nm, 50 mW). ^***^P < 0.001. **g** In vitro phototoxicity of DS-Ce6 in endothelial cells (ECs), smooth muscle cells (SMCs), and macrophages treated with LPS or LPS with LDL under laser irradiation. **h** Annexin V (green) and DAPI (blue)-stained images of foam cells treated with DS-Ce6 (equiv. 5 μM Ce6) after laser irradiation (670 nm, 50 mW). Scale bar = 50 μm. **i** Immunofluorescence staining of LC3 (green), p62 (red), and DAPI (blue) in foam cells treated with DS-Ce6 (equiv. 5 μM Ce6) after laser irradiation (670 nm, 50 mW). Scale bar = 25 μm. ^*^P < 0.05, ^**^P < 0.01, ^***^P < 0.001

Immunofluorescence (IF) and western blot analyses demonstrated the over-expression of SR-A in lipopolysaccharide (LPS)-activated macrophages and foam cells (Fig. 2a, b). Intracellular uptake of DS-Ce6 was evaluated by incubating with foam cells and activated macrophages for 1 h and then detecting using confocal microscopy. DS-Ce6 was taken up by foam cells (Fig. 2c) and by activated macrophages (Additional file 1: Fig. S2a) in a concentration dependent manner. DS-Ce6 exhibited a significantly higher cellular uptake than free Ce6 in foam cells (Fig. 2d) and in activated macrophages (Additional file 1: Fig. S2b). Receptor blocking experiment showed that the uptake of DS-Ce6 by foam cells and activated macrophages was effectively inhibited when SR-As were pre-blocked by DS (Fig. 2d and Additional file 1: Fig. S2b). These results demonstrated that the uptake of DS-Ce6 by activated macrophages and foam cells was induced by overexpressed SR-A on those cells. Interestingly, activated macrophages and foam cells took up DS-Ce6, while smooth muscle cells and endothelial cells did not (Additional file 1: Fig. S2c), indicating that DS-Ce6 specifically targets SR-A expressing activated macrophages and foam cells.

### In vitro apoptotic cell death and autophagy induction after DS-Ce6 photoactivation

The in vitro phototoxicity of DS-Ce6 within macrophages under laser irradiation was examined using the cell counting kit-8 (CCK-8) assay. Cells treated with DS, Ce6, or DS-Ce6 without irradiation exhibited negligible toxicity (Fig. 2e). However, compared to Ce6, DS-Ce6 with light illumination significantly induced phototoxicity at 5 μM of Ce6 (*P* < 0.001) in foam cells (Fig. 2f) and at 10 μM of Ce6 (*P* = 0.008) in the activated macrophages (Additional file 1: Fig. S2d) due to much higher internalization of DS-Ce6 towards these cells. Meanwhile, DS-Ce6 showed negligible phototoxic effects on ECs and SMCs (Fig. 2g) due to a significantly low cellular uptake of DS-Ce6 towards ECs and SMCs (Additional file 1: Fig. S2c).

Apoptosis induction on activated macrophages and foam cells by DS-Ce6 photoactivation was confirmed with annexin V staining. Apoptosis induction was observed in DS-Ce6 treated two cells at 1 min and 2 h after laser irradiation (Fig. 2h and Additional file 1: Fig. S2e; *P* < 0.001). Next, DS-Ce6 photoactivation induced autophagy flux was further evaluated in activated macrophages and foam cells using immunofluorescence staining. DS-Ce6 photoactivation increased the number of LC3 puncta, an indicator of autophagosome formation, within the activated macrophages 15 min after laser irradiation (Additional file 1: Fig. S2f; *P* < 0.001). The number of LC3 puncta peaked at 30 min (Additional file 1: Fig. S2f; *P* < 0.001) and then declined at 1 h (*P* < 0.001). The amount of autophagosome was markedly increased at 30 min in foam cells (Fig. 2i; *P* < 0.001) and then decreased at 1 h (*P* < 0.001), indicating that DS-Ce6 photoactivation induced autophagic flux in activated macrophages and foam cells. These data demonstrated that light activation of DS-Ce6 effectively induces apoptosis and autophagic flux in activated macrophages and foam cells.

### In vivo whole-body imaging and accumulation of DS-Ce6 in atherosclerotic plaques

**Fig. 3:**
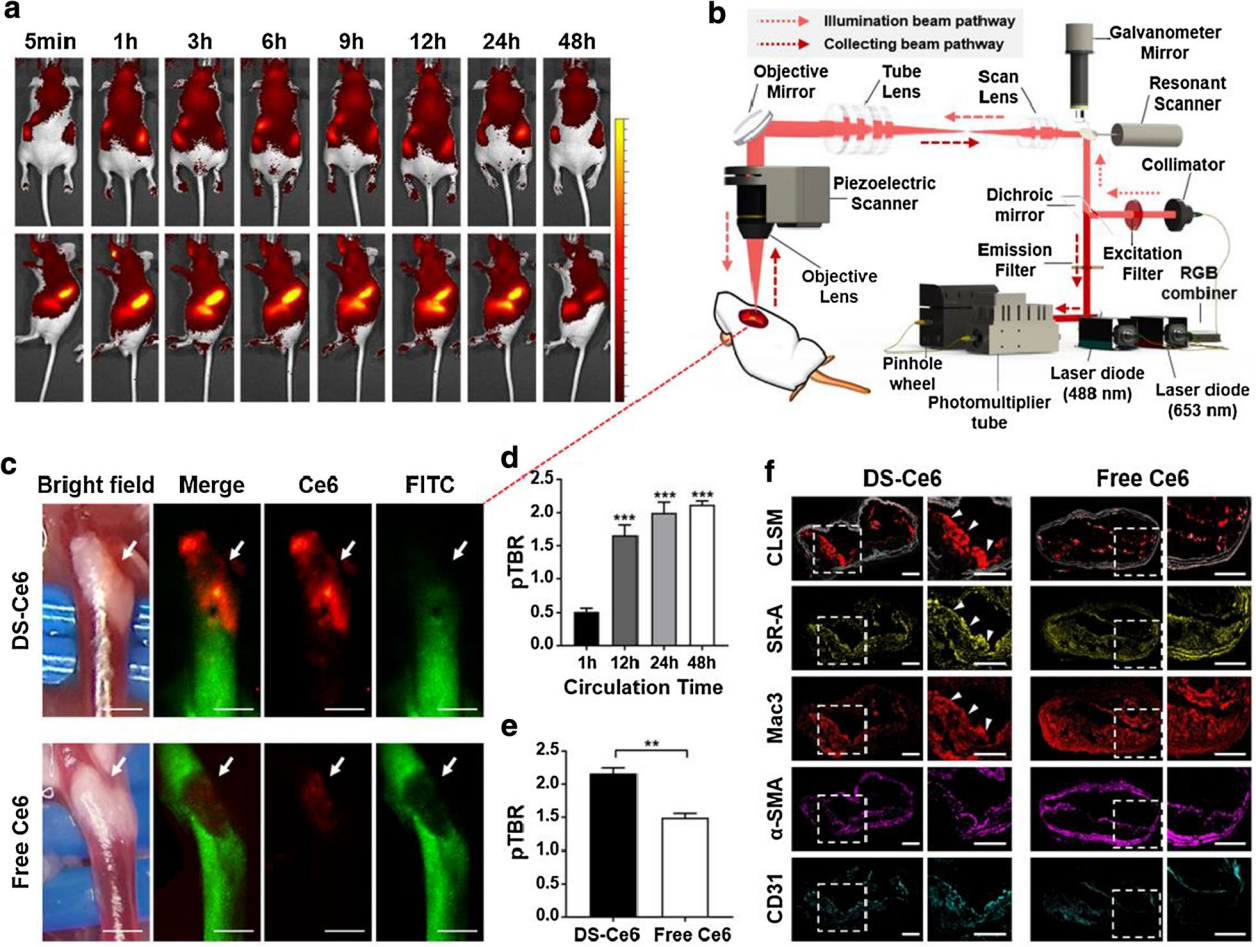
In vivo optical imaging of DS-Ce6 in the carotid plaques of mice with atherosclerosis. **a** In vivo fluorescence imaging of BALB/c nude mice with intravenous injection of DS-Ce6 at different time points (n = 3). **b** Schematic diagram of the custom-built multi-channel IVFM. **c** Representative in vivo imaging of the carotid atheroma at 48 h after the intravenous injection of DS-Ce6 or free Ce6 (intravenous dose: equivalent of 2 mg/kg of Ce6). The fluorescence image in the Ce6 channel (red) demonstrates the DS-Ce6 uptake in a carotid plaque. The FITC angiogram (green) outlines the vasculature. Arrow indicates carotid bifurcation. Scale bar = 400 μm. **d** pTBR of the carotid atheroma that received the intravenous injection of DS-Ce6 according to the circulation time as assessed by IVFM imaging (n = 3 per group). ^***^P < 0.001 versus the pTBR_1h_. **e** pTBR comparison between the DS-Ce6 and free Ce6 injection groups. ^*^P < 0.05. **f** Localization of DS-Ce6 to macrophage SR-A in a carotid atheroma. CLSM image of DS-Ce6 deposition (red) in an atheroma, with elastin autofluorescence (gray). IF staining of the SR-A, macrophages (Mac3), SMCs (α-SMA), and ECs (CD31). CLSM exhibits DS-Ce6 accumulation within the atheroma, which co-localizes with the macrophages and SR-A (white arrowhead). Scale bar = 100 μm

Whole-body fluorescence imaging was obtained over 48 h to monitor the clearance rate of DS-Ce6. Strong fluorescence signal was observed from 1 to 12 h after a single injection of DS-Ce6 and gradually decreased thereafter (Fig. 3a and Additional file 1: Fig. S3a). DS-Ce6 uptake in carotid plaque was evaluated using a custom-built intravital fluorescence microscopy (IVFM) imaging system as described in the Supplementary materials (Fig. 3b, c) [36, 37], which was expressed as a plaque target-to-background ratio (pTBR). To optimize the imaging time, atherogenic mice underwent intravital imaging 1, 12, 24, and 48 h after DS-Ce6 injection. By IVFM imaging, DS-Ce6 accumulated within the plaque 12 h after administration, and the plaque signals were the highest at 48 h (Fig. 3d). However, free Ce6 showed much weaker NIRF signals in the plaque areas of atherogenic mice at 48 h post-injection compared to the DS-Ce6 treated (Fig. 3c and e). Moreover, DS-Ce6 is more specifically targeted and accumulated within the plaques than free Ce6 (confocal laser scanning microscopy (CLSM) in Fig. 3f and Additional file 1: Fig. S3b). IF analysis revealed that Mac3- and SR-A-positive cells co-localized well with the fluorescence signals of DS-Ce6 within the lesions and in contrast, no association was found between the DS-Ce6 fluorescence signals vs. other cells such as SMCs and ECs (Fig. 3f). CLSM and immunohistochemistry (IHC) analyses further supported that macrophages in the atheroma took up DS-Ce6, while SMCs and ECs did not (Additional file 1: Fig. S3c). These data suggest that DS-Ce6 is more effectively delivered into atheroma than free Ce6 via SR-A specific binding, and thus the fluorescence signals of DS-Ce6 in atheroma are highly associated with the inflammatory activity due to its much higher specificity towards macrophages than normal vascular cells.

### In vivo phototherapeutic effects of DS-Ce6

**Fig. 4:**
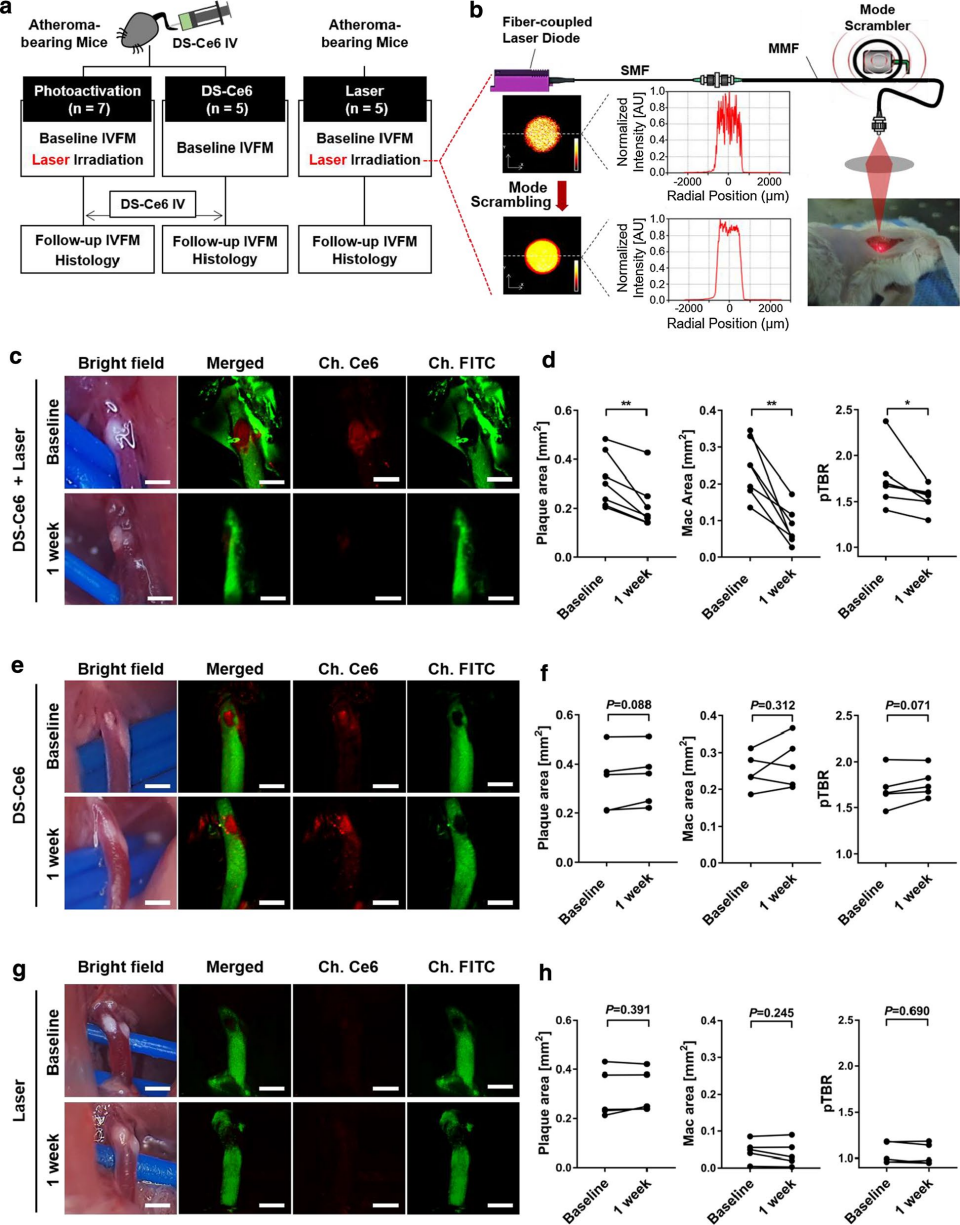
In vivo theranostic effects of DS-Ce6 photoactivation on plaque burden and inflammation. **a** In vivo study protocol of the IVFM-assisted photoactivation and serial imaging of the carotid plaques in the atherogenic mice. **b** Schematic illustration of our custom-built laser modulation system. Mode scrambling technique generates a homogeneous beam profile. **c** Representative serial in vivo plaque imaging at baseline and 1 week after laser irradiation in the DS-Ce6 photoactivation group. **d** Quantitative analysis of the plaque area, macrophage area, and pTBR before and after photoactivation. n = 7 mice, ^*^P < 0.05, ^**^P < 0.01. **e** In vivo optical imaging of the carotid plaques at baseline and 1-week follow-up in the control group administered with DS-Ce6 only. **f** Quantitative analysis in the DS-Ce6 only control group without laser irradiation. ^**^P < 0.01. n = 5 mice. **g-h** Serial in vivo imaging of carotid atheroma (**g**), and quantitative analysis (**h**) in laser irradiation group without DS-Ce6 administration. n = 5 mice

To assess in vivo phototherapeutic effects of DS-Ce6 on atherogenic mice, serial IVFM imaging was performed over a period of 7 days, at baseline and 1 week after laser irradiation (Fig. 4a) using a customized laser system that enables a focal irradiation to the target lesions with a homogeneous beam profile (Fig. 4b) as described in Supplementary materials [38]. Mice received DS-Ce6 at 48 h before in vivo imaging. To look into non-photoactivatable effects of DS-Ce6 on atheroma, atherogenic mice without laser irradiation were compared as controls. In laser irradiated atherogenic mice, the macrophage areas and pTBR decreased significantly by 57.6% (*P* = 0.003) and 10.3% (*P* = 0.044) compared to the pre-photoactivation baseline (Fig. 4c, d). Of interest, the plaque area was also reduced prominently by 29.2% (*P* = 0.004) as compared to the baseline (Fig. 4c, d). In the non-irradiation control mice, plaque area, macrophage area and pTBR did not show significant differences compared to the baseline (Fig. 4e, f). To confirm that the phototherapeutic effects was mediated by DS-Ce6 administration, atherogenic mice received saline injection, and serial IVFM imaging was conducted before and 1 week after laser irradiation. In the absence of DS-Ce6 administration, NIRF signal was not detected in the carotid plaques, and no significant differences were found in plaque area, macrophage area and pTBR between baseline and 1 week follow-up (Fig. 4g, h). These in vivo findings suggest that DS-Ce6 photoactivation could regress the plaque burden and also reduce the inflammation in atherosclerotic plaques at 1 week after laser irradiation.

**Fig. 5:**
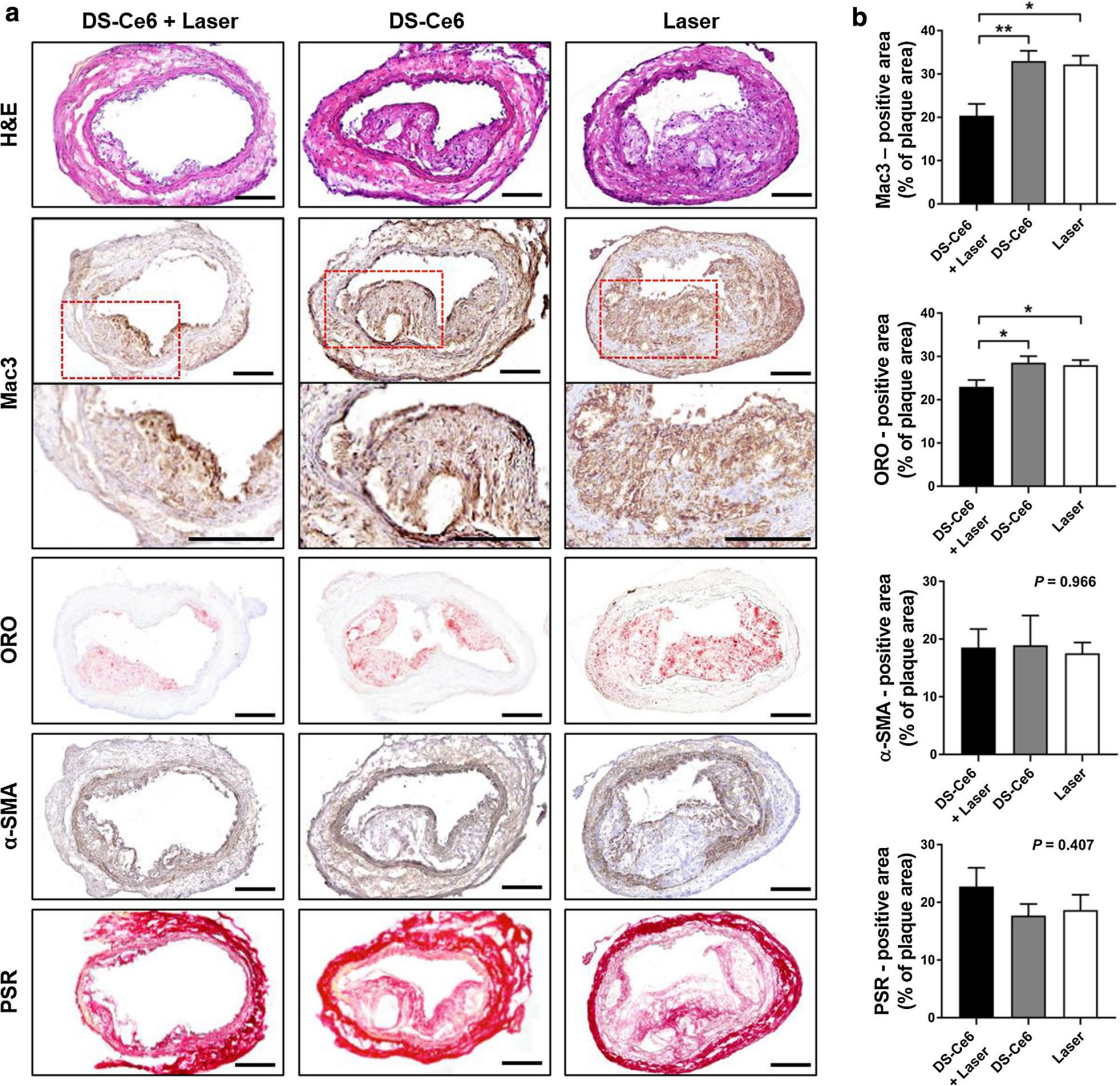
Histological validation of the effects of DS-Ce6 photoactivation on plaque compositions. **a** Representative images of the H&E, ORO and PSR staining, and Mac3 and α-SMA IHC. Scale bar = 100 μm. **b** Quantitative analysis of the Mac3-, ORO, α-SMA-, and PSR-positive areas per plaque area. ^*^P < 0.05

Next, we estimated the phototherapeutic effects of DS-Ce6 on plaque composition by histological analysis of the imaged carotid plaques. In the DS-Ce6 photoactivation-treated mice, the percentage of Mac3 (macrophages) positive area was prominently decreased by 36.7% (*P* = 0.011) and 38.2% (*P* = 0.030) compared to the laser only and non-irradiation control group, respectively (Fig. 5a, b). Oil red O (ORO; lipid) positive area was significantly reduced in DS-Ce6 with laser irradiation group by 17.8% (*P* = 0.021) compared to laser only group, and by 19.5% (*P* = 0.035) compared to the DS-Ce6 only controls. No significant difference in smooth muscle cell contents (α-SMA) was observed across the groups (*P* = 0.966) (Fig. 5a, b). Picrosirius Red (PSR) staining showed that DS-Ce6 photoactivation tended to increase collagen contents within the carotid plaques compared to the laser only (21.9% increase; *P* = 0.562) and DS-Ce6 only group (28.4% increase; *P* = 0.425), although this effect did not reach statistical significance (Fig. 5a, b). Overall, these data indicate that DS-Ce6 photoactivation was able to effectively attenuate macrophage-mediated pro-inflammatory responses and likely shift the plaque composition to favorably stable phenotype in atheroma one week after phototherapy.

### Autophagy induction and efferocytosis enhancement by photoactivation

**Fig. 6:**
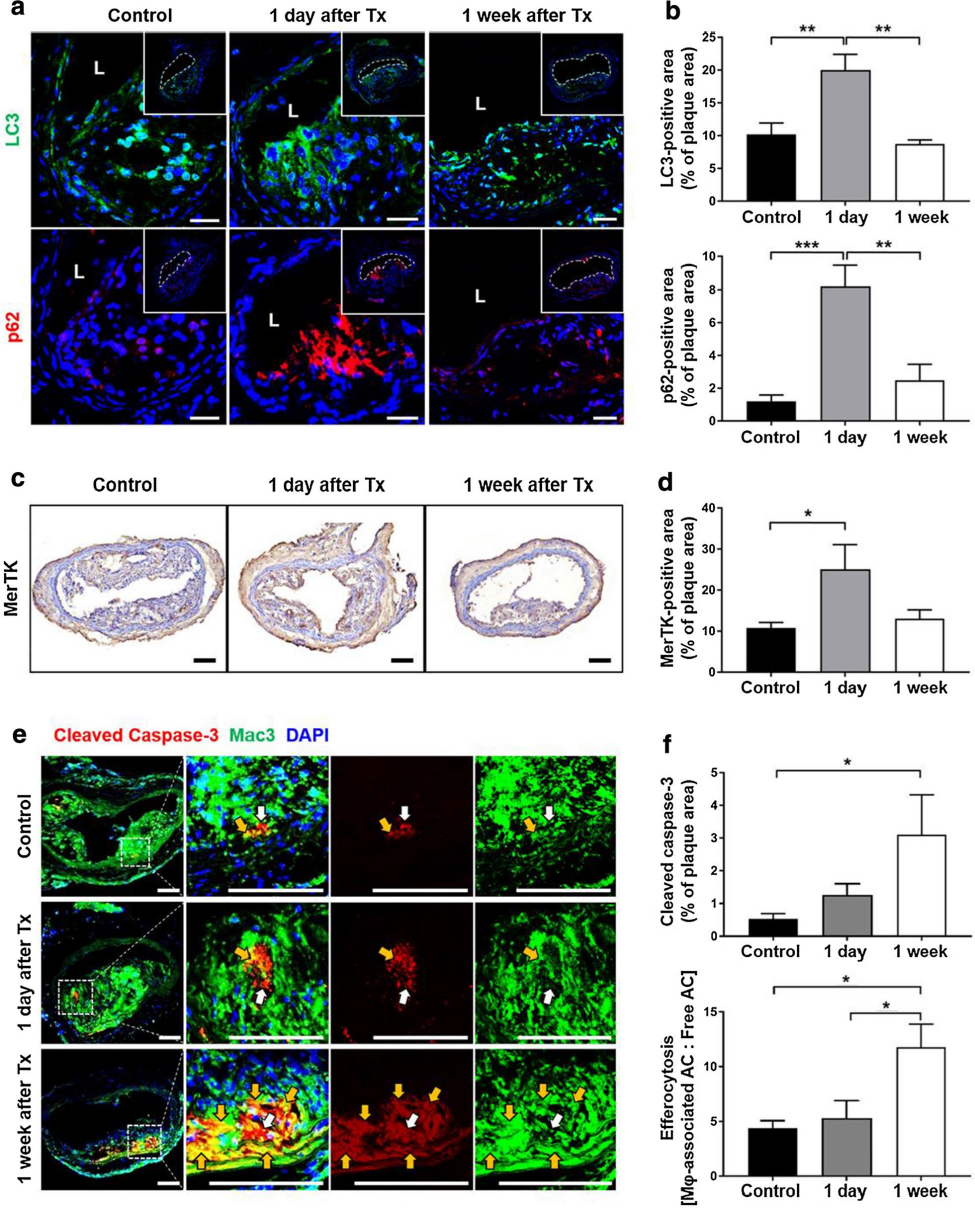
Autophagy activation and efferocytosis enhancement in advanced atheroma over time after photoactivation. **a** CLSM images of the double IF of autophagy marker LC3 (green) and p62 (red) at 1 day and 1 week after laser irradiation. Blue: nucleus stained with DAPI. Scale bar = 100 μm. **b** Quantification of the LC3- and p62-positive areas per plaque. ^**^P < 0.01, ^***^P < 0.001. **c** IHC analysis of MerTK expression in the plaques of the DS-Ce6-photoactivated mice and controls. Scale bar = 100 μm. **d** Quantification of MerTK expression per plaque area. ^*^P < 0.05. **e** Representative fluorescence micrographs of the carotid plaques double-stained with cleaved caspase-3 (red) and Mac3 (green). Scale bar = 100 μm. **f** Cleaved caspase-3 expression per plaque area and efferocytosis quantified as macrophage-associated (yellow arrows) versus macrophage-free apoptotic cells (white arrows) in the plaques after DS-Ce6 photoactivation. ^*^P < 0.05. *Tx* treatment, *Mφ* macrophage, *AC* apoptotic cells

To further determine whether DS-Ce6 photoactivation affects autophagic activity in vivo, we assessed the expression of markers of autophagy progression (LC3) and autophagy chaperone (p62) [39] within atherosclerotic plaques at 1 day and 1 week after laser irradiation. LC3-positive expression per plaque area increased significantly in DS-Ce6 injected mice at 1 day after light activation as compared to the non-irradiated plaques in control mice (Fig. 6a, b; *P* = 0.003). This increase was returned to near control level at 1 week (*P* = 0.001). Similarly, the expression of p62 in atheroma was significantly higher at 1 day after light activation compared to control mice (Fig. 6a, b; *P* < 0.001) and subsequently decreased at 1 week (*P* = 0.002). These time dependent dynamic changes of the autophagy markers indicate immediate induction and early completion of autophagy flux in the atherosclerotic plaque by DS-Ce6 photoactivation.

Next step, to explore whether DS-Ce6 photoactivation enhances efferocytosis in the plaque, we measured the expression of Mer tyrosine kinase (MerTK), a cell surface receptor that promotes internalization of dead cells [40, 41], in irradiated murine atheroma. Immunohistochemical analysis revealed an enhanced MerTK expression at 1 day compared to the controls (Fig. 6c, d; *P* = 0.042). This expression tended to decrease at 1 week. To further assess the interaction of efferocytosis with macrophages, we analyzed the expression of both cleaved caspase-3 and macrophage in the plaque sections. In DS-Ce6 phototreated plaques, the cleaved caspase-3 expression tended to increase at 1 day and was prominent at 1 week after laser irradiation compared to non-treated plaques (Fig. 6e, f; *P* = 0.038). To determine whether DS-Ce6 photoactivation-induced apoptotic cells are associated with enhanced efferocytosis, we compared the number of macrophage-associated apoptotic cells to free apoptotic cells. In the control group, free apoptotic bodies in plaques were observed as bright red-colored areas that did not overlap with the macrophages (white arrows in Fig. 6e). At 1 week after DS-Ce6 photoactivation, efferocytosis significantly increased by 2.7-fold compared to the control (*P* = 0.014), and 2.2-fold compared to day 1 (*P* = 0.016) (Fig. 6e, f). These findings altogether indicate that light activation of DS-Ce6 induces autophagy and MerTK upregulation at an early time point, further promoting efferocytosis of apoptotic cells.

## Discussion

Inflammatory cell is a major contributor in all stages of atherosclerosis including catastrophic sudden plaque rupture [5, 16]. Growing evidence suggests that macrophage apoptosis and subsequent clearance of the dead cells are crucial for the resolution of atherosclerosis [42, 43]. Our photoactivation strategy targeting macrophages could in vivo detect the inflammation activity in the atheroma and simultaneously provide robust anti-atherosclerotic effects with macrophage ablation. Following laser irradiation, DS-Ce6 with a strong affinity for SR-A expressing macrophages generated the ROS in the atheroma and also emitted the NIRF signals enabling a serial in vivo assessment of the therapeutic efficacy by a customized intravital imaging. Intriguingly, photoactivation of DS-Ce6 augmented apoptotic cell clearance via autophagy induction with facilitated efferocytosis. Current study provides the evidence to demonstrate net favorable in vivo effects of targeted photoactivation on atheroma, showing stabilization and regression of the inflamed plaque. Furthermore, our results revealed the compelling mechanistic ground for the photoactivation therapy in the atherosclerosis.

The advent of photoactivation represents a promising alternative to locally treat the atherosclerosis without any remnants such as metallic stent. In earlier researches using dextran-coated or cathepsin-B activatable nanoagent, photoactivation failed to achieve a meaningful decrease in atheroma burden [19, 20], whereas they significantly reduced the macrophage contents [20]. By contrast, our DS-Ce6 photoactivator upon laser irradiation provided a robust reduction of both plaque burden and macrophage contents. This unique anti-atherogenic effects of current DS-Ce6 photoactivation is probably attributable to the specific binding affinity of DS-Ce6 to the macrophages, high-dose photosensitizer administration, and irradiance enhancement with laser beam homogenization, which altogether enables a full activation of photosensitizers in the plaques. In the present study, we newly fabricated a photoactivatable nanoagent through conjugation of Ce6, NIRF-emitting photosensitizers, to DS as a target ligand for SR-A [32] which is highly expressed in activated macrophage and foam cell [33]. DS-Ce6 formed a self-assembled nanostructure and could target macrophages via SR-A-mediated endocytosis, exhibiting a prolonged circulation time [44]. Consequently, the binding affinity of DS-Ce6 specific to the macrophages was substantially improved. The damage to SMCs and ECs could render the plaque more prone to rupture and thus should be minimized [16, 17, 45]. In current study, by a single irradiation of laser with targetable bandpass, any damages to other vascular structures were not evident. The specific binding property of DS-Ce6 to the target cells could avoid the non-specific deleterious effects on the lining ECs and SMCs of the plaque. In addition, a uniform laser intensity directed at the target lesion by applying mode scrambling in combination with a plano-convex lens likely contributes to the net therapeutic benefits of photoactivation.

While a potent photoactivation was to reduce plaque macrophage contents more effectively via apoptosis induction [19, 20], unfortunately, these benefits might be offset by increase of necrotic core in the plaque. As efferocytosis is defective in advanced atheroma, abundant apoptotic materials are potentially pro-inflammatory and even likely grow the necrotic core [21, 46, 47]. Given a concern regarding detrimental effects of apoptosis on necrotic core, the apparent capability of light-activated DS-Ce6 to reduce both plaque burden and inflammation is particularly intriguing. While apoptosis causing macrophage depletion has been suggested as the main cellular mechanisms for the therapeutic effects of photoactivation on atherosclerotic plaques [9, 18–20], the earlier in vitro studies have shown that photoactivation not only induces apoptosis but also increases autophagic activity [23, 48]. Moreover, upconversion nanoparticle-mediated photoactivation enhances autophagy and cholesterol efflux in macrophage-derived foam cells [25]. However, whether photoactivation could effectively induce autophagy in atherosclerosis in vivo and its ultimate effects on the treated atheroma are still questionable. In the present study, using the common markers of autophagic activity (LC3) and autophagy chaperone (p62) [39], photoactivation with DS-Ce6 was shown to induce autophagic activity in murine atheroma. Since the autophagy enhances efferocytosis by promoting the recognition of apoptotic cells and degradation of phagocytosed cargo [26–28, 49], we further investigated the expression of efferocytosis receptor MerTK and efferocytosis capacity over time after DS-Ce6 activation. Interestingly, activated DS-Ce6 enhanced the clearance of apoptotic cells after autophagy activation and MerTK upregulation. Taken altogether, harmonized augmentation of autophagy and efferocytosis following photoactivation appears to ameliorate the potential harmful effects of macrophage apoptosis on atheroma.

Our study has some limitations. First, we applied a 670 nm laser power of 1.0 W/cm^2^ which was relatively higher than those used in previous studies [9, 19, 20]. In a recent first-in-man NANOM trial, applied 35–44 W/cm^2^ laser irradiation power for 7 min could bring the photothermal effects on atherosclerosis, while the degree of photoenergy utilized in the current study was considered to be acceptable in vivo [50]. Second, autophagy in atherosclerosis is commonly associated with enhanced efferocytosis [26–28], however, here we could not show the biological interconnections with respect to photoactivation. Additional mechanistic studies are required to clarify the linked molecular pathways involved in the process. Finally, it is unclear whether the beneficial efficacy of photoactivation is specific to current targeted agent. Further studies for the applicability to other type of photoactivatable agents will be needed.

The clinical implementation of DS-Ce6 photoactivation is relevant to preemptive treatment of coronary artery disease. Based on our findings, DS-Ce6 photoactivation seems to rapidly suppress plaque inflammation and even regress atheroma burden by macrophage apoptosis and its effective clearance via autophagy activation with enhanced efferocytosis. When applied to coronary artery disease, innovative NIRF catheter imaging-assisted photoactivation of DS-Ce6 through integrated fiber-based laser irradiation could be a promising theranostic strategy for simultaneous assessment and treatment of inflamed high-risk plaque in coronary arteries [37, 51, 52].

## Conclusions

The novel macrophage SR-A-targeted theranostic agent strongly accumulated within inflamed atherosclerotic plaques. NIRF imaging-guided photoactivation was able to reduce plaque inflammation and burden via autophagy induction with enhanced efferocytosis (Scheme 1). These results offer molecular evidence to support the photoactivation treatment in atherosclerosis and could open a new avenue for imaging-assisted theranostic strategy for high-risk plaques.

## Methods

### Materials

DS (MW = 40,000 Da), CDI, anhydrous dimethyl sulfoxide (DMSO), LDL, and LPS were purchased from Sigma-Aldrich (St. Louis, MO, USA). Ce6 was purchased from Frontier Scientific (Logan, UT, USA). Singlet oxygen sensor green (SOSG) and 4, 6-diamidino-2-phenylindole dihydrochloride (DAPI) were obtained from Invitrogen (Carlsbad, CA, USA). The Apoptosis/Necrosis Detection Kit was purchased from Abcam (Cambridge, UK).

### Preparation and characterization of DS-Ce6

Macrophage targetable phototheranostic nanoagent (DS-Ce6) was synthesized as follows: DS (500 mg) was dissolved in 20 mL of anhydrous DMSO at 70 ℃ for 2 days under nitrogen. Ce6 (100 mg) and CDI (57 mg) were reacted in 15 mL of anhydrous DMSO for 6 h to activate Ce6. The activated Ce6 solution was added to the DS solution and reacted at 70 ℃ for an additional 2 days. The reaction mixture was precipitated in cold acetone and washed four times to remove CDI and unreacted Ce6. After removing the acetone in vacuum, the dried DS-Ce6 was dispersed in deionized (DI) water and lyophilized for 2 days.

For ultraviolet/visible (UV/Vis) spectroscopic analysis, DS-Ce6 (1 mg) was completely dissolved in 1 mL of 0.1 M NaOH/0.1% sodium dodecyl sulfate (SDS; sigma). The standard solutions of Ce6 with different concentrations were also prepared using 0.1 M NaOH/0.1% SDS solution. The number of conjugated Ce6 molecules to DS was calculated using the standard curve of Ce6 (Y = 0.2924x + 0.02 [R^2^ = 0.9995]), which was obtained by measuring the absorbance at 400 nm using UV-1800 UV/Vis spectrophotometer (Shimadzu, Kyoto, Japan). The synthesis of DS-Ce6 was confirmed using Fourier transform infrared spectroscopy (FT-IR; Shimadzu 8400S, Kyoto, Japan). Using the KBr pellet method, the FT-IR spectra of DS-Ce6 and pure DS were recorded at a resolution between 4000 and 400 cm^−1^.

To determine the particle size and morphology, DS-Ce6 (0.5 mg) was dispersed in DI water (1 mL) and the particle sizes were measured using a SZ-100 particle size analyzer (HORIBA, Kyoto, Japan). To test the stability of DS-Ce6, DS-Ce6 (1.5 mg) was dispersed well in 3 mL of PBS (pH 7.4) or 10% FBS-containing Dulbecco's Modified Eagle Medium (DMEM, Welgene, Seoul, Korea) using probe-type sonicator and then their hydrodynamic diameters were monitored for 7 days. The morphology of DS-Ce6 was determined by TEM (LEO 912AB OMEGA, Carl Zeiss, Oberkochen, Germany). Particle sizes in TEM images were determined using ImageJ software.

To confirm the self-assembly of DS-Ce6 in aqueous condition, DS-Ce6 was dispersed in PBS. For comparison, free Ce6 and DS-Ce6 were solubilized in PBS containing Tween 20 (1%, v/v). The final concentration of each solution was set to equivalent dose of 5 μM for Ce6. UV/Vis spectra of the two groups were obtained in the 300–800 nm range using a UV/Vis spectrophotometer. The fluorescence spectra of the three groups were measured using a model FS-2 fluorescence spectrophotometer (Scinco, Seoul, Korea) at 550–800 nm with a 670 nm excitation wavelength. The fluorescence images of three groups were acquired using an IVIS 200 imaging system (Xenogen Corp., Alameda, CA, USA).

The generated ^1^O_2_ levels were evaluated by measuring the fluorescence intensity of SOSG (Invitrogen, Carlsbad, CA, USA) under light illumination. DS-Ce6 (equiv. 5 μM Ce6) was dispersed in oxygen-saturated PBS. For comparison, free Ce6 (5 μM) and DS-Ce6 were dissolved in oxygen-saturated PBS containing Tween 20 (1% v/v). SOSG reagent (1 μM) was added to the three groups, and ^1^O_2_ generation was recorded by measuring the fluorescence intensity of SOSG (Ex/Em 504/525 nm) using a model FS-2 fluorescence spectrophotometer (Scinco) under light irradiation (670 nm continuous wave laser, 50 mW/cm^2^).

### SR-A expression in LPS-activated macrophages and macrophage foam cells

The expression of SR-A in LPS-activated macrophages and macrophage foam cells was evaluated using western blot analysis and IF staining. For western blot analysis, RAW264.7 macrophages (Korean Cell Line Bank, Seoul, Korea) were incubated in RPMI 1640 medium (Welgene, Gyeongsan, South Korea) supplemented with 10% FBS, 100 U/mL penicillin, and 100 μg/mL streptomycin at 37 °C in a humidified 5% CO_2_ atmosphere. Macrophages were seeded at 1 × 10^5^ cells in each 60 mm dish and allowed to adhere for 24 h. The cells were treated with LPS (200 ng/mL) for 24 h to activate macrophages or with LPS in the presence of LDL (100 μg/mL) to induce macrophage foam cells. Cells were then lysed with M-PER Mammalian Protein Extraction Reagent (Thermo Fisher Scientific, Waltham, MA, USA). The cell lysates were collected by centrifugation at 14,000 rpm for 30 min at 4 °C and stored at − 80 °C. The cell lysates (80 μg/lane) were separated by 10% sodium dodecyl sulfate–polyacrylamide gel electrophoresis (SDS-PAGE) and transferred to polyvinylidene difluoride membranes. The membranes were immunoblotted with a primary antibody specific for SR-A (1:500; Novus Biologicals, Centennial, CO, USA) at 4 °C overnight, incubated with a secondary antibody for 1 h at room temperature, and then detected using a chemiluminescence western blotting detection kit (Bio-D, Gwangmyeong, Korea). Cofilin was used as loading control. Quantification of western blot was performed using ImageJ, normalized to loading control, and presented as fold change over unstimulated controls.

For the IF assay of SR-A expression, RAW264.7 cells (5 × 10^5^ cells per plate) were seeded in 8-well chamber slides (SPL Life Sciences, Gyeonggi‐do, Korea), incubated for 24 h, and then stimulated with LPS and LDL for 24 h. The cells were fixed with 4% paraformaldehyde in PBS (pH 7.4) for 10 min at room temperature, blocked with 1% bovine serum albumin (BSA) for 30 min, and incubated with an SR-A antibody (1:100; Novus Biologicals) for 2 h at room temperature, followed by incubation with Alexa Fluor 488 AffiniPure goat anti-rabbit IgG secondary antibody (1:100; Jackson ImmunoResearch, West Grove, PA, USA) for 45 min at room temperature. After washing with PBS, the cells were mounted using a fluorescence mounting medium with DAPI (GBI Labs, Bothell, WA, USA) and examined using an LSM 780 Meta NLO confocal microscope (Carl Zeiss). The SR-A expression was quantified as the percentage of positively stained cells per at least 20 cells (n = 5).

### Intracellular uptake of DS-Ce6 and blocking experiments

RAW264.7 cells (5 × 10^5^ cells per plate) were seeded in 8-well chamber slides and allowed to adhere for 24 h. Cells were stimulated with LPS or LPS in the presence of LDL for 24 h and maintained in a serum-free medium containing various concentrations of DS-Ce6 (equivalent doses of 0, 1, 5, and 10 μM Ce6) for 30 min. To compare the intracellular uptake of free Ce6, cells were incubated with free Ce6 (5 μM) for 30 min. For blocking experiments, activated macrophages and foam cells were incubated with a serum-free medium containing free DS (5 mg/mL) as a SR-A ligand for 1 h, and then treated with DS-Ce6 (equiv. 5 μM Ce6) for 1 h. After washing with PBS (pH 7.4), the cells were fixed with 4% paraformaldehyde for 30 min, washed twice with PBS, and mounted on a slide glass. The intracellular uptake and blocking experiments were examined using a customized CLSM (Ex/Em: 633 nm/650–710 nm; see Multichannel confocal laser scanning IVFM system section in Supplementary Information). For each data point, the mean fluorescence intensity of at least 20 cells was quantified using ImageJ software (n = 5).

To compare the intracellular uptake of DS-Ce6 within various vascular cells, mouse endothelial cells (ECs; ATCC, Manassas, VA, USA) (5 × 10^4^ cells per well), smooth muscle cells (SMCs; ATCC) (5 × 10^4^ cells per well), and RAW264.7 cells (1 × 10^5^ cells per well) were seeded in 8-well chamber slides and incubated for 24 h. RAW264.7 cells were stimulated with LPS or LPS with LDL for 24 h to activate or to induce foam cells, respectively. Cells were treated with DS-Ce6 (equivalent dose of 5 μM) for 1 h, washed thrice with PBS, and then fixed with 4% paraformaldehyde for 15 min. After washing and mounting, the cells were imaged with our customized confocal laser scanning microscopy system (Ex/Em: 633 nm/650–710 nm) as described in the Supplementary Information. For each data point, the mean fluorescence intensity of at least 20 cells was quantified using ImageJ software (n = 5).

### In vitro phototoxic effects of DS-Ce6 photoactivation on vascular cells

To investigate the cell viability following DS-Ce6 and laser treatment, cell viability was measured using the CCK-8 assay (Dojindo Laboratories, Kumamoto, Japan) according to the manufacturer’s instructions. The RAW264.7 cells were seeded in black 96-well plates at a density of 2 × 10^4^ cells per well and cultured for 24 h to adhere. Thereafter, they were treated with LPS (200 ng/mL) to activate the cells or LPS in the presence of LDL (100 μg/mL) to induce foam cells for 24 h. The medium was replaced with a serum-free medium containing DS (0, 1, 2, 5, 10, and 20 μM), free Ce6 (0, 1, 2, 5, 10, and 20 μM), or DS-Ce6 (equivalent doses of 0, 1, 2, 5, 10, and 20 μM Ce6), and the cells were maintained for 1 h in the dark. After washing with PBS, the cells in the phototoxicity group were irradiated using a 670 nm laser (50 mW, irradiated area 3.8 cm^2^) for 10 s, and the cells in the dark toxicity group were not irradiated with laser. After 24 h, 20 μL of CCK-8 solution was added to each well with 200 μL of medium and the cells were further incubated for 1 h. The optical density (OD) values for each well were read using a microplate reader at a wavelength of 450 nm. All experiments were conducted in triplicate. Cell viability was defined as the percentage of living cells per that of the control cells at 0 μM.

To compare the phototoxicity in various vascular cells, ECs, SMCs, activated macrophages, and foam cells were treated with DS-Ce6 (equivalent doses of 0, 2, and 5 μM Ce6) for 1 h. The cells were irradiated with a 670 nm laser (50 mW, irradiated area 3.8 cm^2^) for 10 s and further incubated in the dark for 24 h. The CCK-8 assay was performed as previously described, and the OD values for each well were measured at 450 nm. All experiments were conducted in triplicate, and cell viability was defined as the percentage of viable cells per total control cells at 0 μM.

### Apoptosis assay

RAW264.7 macrophages (5 × 10^5^ cells/well) were seeded and stimulated with LPS or with LPS in the presence of LDL for 24 h. They were then treated with DS-Ce6 (equiv. 5 μM Ce6) for 1 h and the medium was replaced with fresh cell culture medium. The cells were irradiated using a 670 nm laser (50 mW, irradiated area 3.8 cm^2^) for 10 s and further incubated for 1 min and 2 h. For comparison, non-irradiated cells were prepared. Apoptosis induction was detected using a commercial Apoptosis/Necrosis Detection Kit (Abcam). The fluorescence signal was examined using a model LSM 780 Meta NLO confocal microscope (Carl Zeiss). The rate of apoptosis was quantified as the percentage of annexin V-positive cells per at least 40 cells (n = 5) [53].

### Autophagy assay

RAW264.7 cells (5 × 10^5^ cells) were seeded and incubated with LPS or LPS in the presence of LDL. The cells were treated with DS-Ce6 (equiv. 5 μM Ce6) for 1 h, irradiated using a 670 nm laser (50 mW, irradiated area 3.8 cm^2^) for 10 s, and further incubated for 15 min, 30 min, and 1 h. Cells without laser irradiation were used as a control. The cells were fixed with 4% paraformaldehyde in PBS (pH 7.4) for 10 min at room temperature, blocked with 1% BSA for 30 min, and incubated with LC3 antibody (1:200; Novus Biologicals) for 1 h at room temperature, followed by incubation with Alexa Fluor 594 AffiniPure goat anti-rabbit IgG (1:100) in the dark. After washing three times with PBS for 5 min each in the dark, the cells were incubated with p62 antibody (1:200; Abcam) for 1 h at room temperature and then with Alexa Fluor 488 AffiniPure goat anti-rabbit IgG (1:100) in the dark. After washing with PBS, the cells were mounted using a fluorescence mounting medium with DAPI. Fluorescence was observed using a model LSM 780 Meta NLO confocal microscope (Carl Zeiss). The LC3 puncta per cell of at least 20 cells in each experimental arm was counted by a blinded observer (n = 5).

### In vivo whole-body biodistribution

Three 7-week-old male BALB/c nude mice (DooYeol Biotech, Seoul, Korea) were intravenously injected with DS-Ce6 (equivalent dose of 0.5 mg/kg Ce6), and whole-body fluorescence imaging was performed in the prone and lateral positions at 5 min and 1, 3, 6, 9, 12, 24, and 48 h post-injection to monitor the in vivo whole-body biodistribution of DS-Ce6 using a small animal imaging system (IVIS 200). Whole-body fluorescence intensities were quantified using ImageJ software.

### IVFM imaging and histological validation for localization of DS-Ce6 to plaque macrophages via SR-A targeting

Male spontaneously hyperlipidemic apolipoprotein E (ApoE) deficient mice with a genetic background of BALB/c (BALB/c.KOR/StmSlc-Apoe^*shl*^) were purchased from a Japan SLC (Shizuoka, Japan) [54]. Previous studies have reported that the ApoE deficient mice develop atheroma in widespread regions including aortic root, principal branches of the aorta, and pulmonary and carotid arteries [54, 55]. In this study, we imaged carotid plaques of atherogenic mouse model using customized IVFM and macrophage targeted photoactivatable agent, which enabled dynamic and longitudinal investigations of plaque characteristics, including lesion size and macrophage activity in vivo [56]. To confirm the DS-Ce6 uptake in the plaque macrophages and localization to the inflamed atherosclerotic plaques, atherosclerosis-prone mice at 7 weeks of age were fed an atherogenic diet (Research Diets, Inc., New Brunswick, NJ, USA) for 10 weeks. The mice received an intravenous injection of DS-Ce6 (equivalent dose of 2 mg/kg of Ce6), the optimum dose to detect the fluorescence signal of DS-Ce6 in carotid plaques, at 1, 12, 24, or 48 h before imaging (n = 3 per group). Thereafter, they were anesthetized with 1.5% isoflurane and the carotid artery was surgically exposed with placement of a 0.5 mm fiber under the vessel to separate it from the adjacent tissues and to reduce the beating motion artifact. Immediately before imaging, fluorescein isothiocyanate (FITC)-dextran (Sigma-Aldrich) was intravenously injected to provide a vascular angiogram outlining the plaque. The experimental animals were imaged using a custom-built IVFM [36, 37] with two laser lines, 488 nm for angiography and 633 nm for DS-Ce6 macrophage activity. The signal of plaque macrophage activity was determined as the mean signal intensity (SI) from the plaque. The plaque area was defined by the filling defect area in the FITC angiogram. The background signal was determined as the mean SIs of the normal artery adjacent to the plaque. For quantitative analysis, the pTBR was calculated as follows: pTBR = [SI (plaque) / SI (adjacent normal artery)]. For a comparison, 2 mg/kg of non-targetable free Ce6 was intravenously injected into the same animal model (n = 3). Forty-eight hours after injection, we performed the IVFM imaging, and the pTBR values were compared between the DS-Ce6 and free Ce6 group.

To determine the targetability of DS-Ce6 in plaque macrophages, IF and IHC analyses were performed. Mice were euthanized, and the brachiocephalic artery and imaged carotid arteries were serially cryosectioned at 10 μm. Custom-built CLSM was used to visualize the distribution of DS-Ce6 within the plaque. The sister sections were then immunostained for the macrophages, smooth muscle cells, and endothelial cells. For IHC analysis, the sections were incubated with Mac3 antibody (1:1000; BD Pharmingen, San Jose, CA, USA), α-smooth muscle actin (α-SMA) antibody (1:100; Abcam), and CD31 (1:100; Novus Biologicals) for 1 h at room temperature. Mac3-, α-SMA-, and CD31-stained areas were detected using the Polink-2 HRP Plus Mouse DAB Detection System (GBI Labs). All slides were dehydrated, cleared, mounted, and observed under a BX51 light microscope (Olympus, Tokyo, Japan). For IF analysis, the cryosections were treated with SR-A antibody (1:100; Novus Biologicals) or α-SMA antibody (1:100) for 1 h at room temperature. After washing with Tris-buffered saline with Tween 20 (TBST) for 30 min, the slides were incubated with Alexa Fluor 488 AffiniPure goat anti-rabbit IgG (1:100) for 1 h in the dark, and then washed three times with TBST for 10 min. Thereafter, the SR-A- or α-SMA-stained slides were incubated with Mac3 (1:100) or ECs (1:25) for 1 h at room temperature, respectively. The slides were then incubated with Alexa Fluor 594 goat anti-rat (1:100; BioLegend, San Diego, CA, USA) or anti-mouse IgG (1:100; BioLegend) for 1 h in the dark. After washing with TBST, the cryosections were mounted using a fluorescence mounting medium with DAPI, and the fluorescence was observed using a Zeiss LSM 780 Meta NLO confocal microscope (Carl Zeiss).

### In vivo imaging-guided photoactivation and histopathological analysis

For imaging-guided photoactivation, seventeen atherogenic mice (7-week-old, male) were fed an atherogenic diet for eight weeks. DS-Ce6 (equivalent dose of 2 mg/kg of Ce6) was administered via intravenous injection, and baseline imaging was performed 48 h after injection using our IVFM system. After baseline imaging, the mice were randomized to either the laser treatment (DS-Ce6 photoactivation, n = 7) or non-irradiated control group (DS-Ce6 only control, n = 5). Mice without DS-Ce6 administration were injected with saline, and used as laser only group (Laser only, n = 5). In the DS-Ce6 photoactivation group, the imaged carotid plaque was externally illuminated using a 670 nm diode laser at a fluence rate of 1 W/cm^2^ for 150 s. The skin of the mice was surgically closed and the animals continued to be fed a normal chow. After 1 week, the same dose of DS-Ce6 was injected, and follow-up imaging was performed in the same carotid atheroma with identical settings to validate the serial changes of the plaque features, including the size of plaque burden and inflammatory activity. Three different atherosclerosis-related parameters were measured: (i) plaque area by FITC angiogram, plaque inflammation by (ii) the macrophage area, and (iii) pTBR. After follow-up IVFM imaging, the mice were euthanized using CO_2_ inhalation and perfused with PBS. The imaged carotid arteries were dissected and snap-frozen for histopathological analysis. All animal studies were approved by the Institutional Animal Care and Use Committee of the Korea University College of Medicine (KOREA-2018–0066, KOREA-2021–0084).

For histopathological analysis, the dissected carotid artery was embedded in an optimal cutting temperature compound (Sakura Finetek, Tokyo, Japan), and serial cryosections were acquired at a thickness of 10 μm. To assess the effects of DS-Ce6 photoactivation on plaque composition, serial cryosections were stained with Hematoxylin and Eosin (H&E; ScyTek Laboratories, Logan, Utah, USA) for morphology, ORO (ScyTek) for lipid accumulation, and PSR (ScyTek) for collagen distribution. Adjacent sections were immunohistochemically stained with Mac3 antibody (1:1000) for macrophages and α-SMA antibody (1:200) for vascular SMCs. The stained slides were captured using a AXIO Scan.Z1 slide scanner (Carl Zeiss). Quantitative values were acquired as the percentage of the positively stained area divided by the plaque area using ImageJ and MATLAB (The Mathworks, Newton, MA, USA).

### Efferocytosis study

For the mechanical study, three atherogenic mice were fed an atherogenic diet for 8 weeks and assigned to the 1 day after treatment group. DS-Ce6 (equivalent dose of 2 mg/kg of Ce6) was intravenously injected into atherogenic mice, and the imaging-assisted photoactivation protocol was applied. After 1 day, the mice were euthanized using CO_2_ inhalation, and the carotid arteries were dissected, snap-frozen, and serially sectioned at a thickness of 10 μm. For IHC and IF analyses, two cryosections were sampled at 150 μm intervals from the carotid bifurcation to the proximal portion.

To validate whether DS-Ce6 photoactivation induced autophagy within the atheroma, IF staining was performed to measure the expression of LC3 and p62. MerTK expression was analyzed by IHC using a MerTK primary antibody (1:300 for 1 h; Santa Cruz Biotechnology). Efferocytosis capacity was assessed by double IF staining of cleaved caspase-3 and macrophages within the same carotid plaques. The expression of cleaved caspase-3 was classified as Mac3-overlapped “macrophage-associated apoptotic cells” and un-overlapped “free apoptotic cells.” Efferocytosis capacity was quantified by the ratio of “macrophage-associated apoptotic cells” to “free apoptotic cells.” For double IF staining, the cryosections were fixed with acetone for 10 min and treated with peroxidase blocking solution for 5 min. The tissue sections were incubated with cleaved caspase-3 antibody (1:400; Cell Signaling Technology, Danvers, MA, USA) overnight at 4 °C. The slides were washed with TBST for 30 min, followed by incubation with Alexa Fluor 594 AffiniPure goat anti-rabbit IgG (1:100) for 1 h in the dark. After washing three times with TBST for 10 min each in the dark, the tissues were incubated with Mac3 antibody (1:100) for 1 h at room temperature, washed with TBST for 30 min, and then treated with Alexa Fluor 488 anti-rat IgG (1:100; BioLegend) for 1 h in the dark. After washing with TBST, the cryosections were mounted using a fluorescence mounting medium with DAPI, and the fluorescence was observed using a CLSM (LSM 780, Carl Zeiss).

### Statistical analysis

Statistical analysis were performed using GraphPad Prism (GraphPad Software, La Jolla, CA, USA) and SPSS (SPSS, Inc., Chicago, IL, USA). The data are expressed as the mean ± standard error of the mean. The paired *t*-test was used to assess the differences in plaque area, macrophage area, and pTBR between the baseline and follow-up imaging. Comparison of normally distributed data were analyzed using the Student’s *t*-test (two groups) or the one-way ANOVA test (three or more groups) with the appropriate post-hoc test. Assumptions of the normality were validated using the Shapiro–Wilk test. Comparison of non-normally distributed data were analyzed using Mann–Whitney U test (two groups) or the Kruskal–Wallis test followed by Dunn’s post-hoc test (three or more groups). *P* values < 0.05 were considered statistically significant. All tests were two-tailed.

## Supplementary Information

**Additional file 1: Figure S1.** FT-IR spectra of (1) DS and (2) DS-Ce6. **Figure S2. a** Representative confocal microscopic images and quantification of dose-dependent cellular uptake of DS-Ce6 in the activated macrophages. ^*^P < 0.05, ^***^P < 0.001. **b** Comparison of the intracellular uptake of free Ce6 (5 μM) and DS-Ce6 (equiv. 5 μM Ce6) in the activated macrophages. To evaluate receptor-mediated endocytosis, the SR-A ligand DS was pre-treated for 1 h before DS-Ce6 incubation (DS + DS-Ce6). ^***^P < 0.001. Scale bar = 50 μm. **c** Comparison of the intracellular uptake of DS-Ce6 (equiv. 5 μM Ce6) in ECs, SMCs, and RAW264.7 macrophages treated with LPS or LPS with LDL. ^***^P < 0.001 compared to ECs, and ^###^*P* < 0.001 compared to SMCs. Scale bar = 30 μm. **d** Viability of activated macrophages treated with different concentrations of DS-Ce6, DS, and Ce6 with laser irradiation (670 nm, 50 mW). ^**^P < 0.01. **e** DS-Ce6 photoactivation-induced apoptosis according to the time after laser irradiation. Annexin V (green) and DAPI (blue)-stained images of the activated macrophages that received DS-Ce6 treatment (equiv. 5 μM Ce6) and laser irradiation (670 nm, 50 mW). ^***^P < 0.001. Scale bar = 50 μm. **f** DS-Ce6 photoactivation-induced autophagy flux according to the time after laser irradiation. IF staining of LC3 (green), p62 (red), and DAPI (blue) in the activated macrophages that received DS-Ce6 treatment (equiv. 5 μM Ce6) and laser irradiation (670 nm, 50 mW). Scale bar = 25 μm. ^***^P < 0.001. **Figure S3. a** Quantification of whole-body fluorescence in the lateral and prone positions at 0 and 5 min and 1, 3, 6, 9, 12, 24, and 48 h post-injection (n = 3). **b** Quantification of Ce6 fluorescence intensity within plaques treated with DS-Ce6 or free Ce6. ^*^P < 0.05. **c** Co-localization of DS-Ce6 (CLSM) and macrophages (Mac3) within the atheroma (red arrow). Scale bar = 100 μm.

## Data Availability

All the original data are available upon reasonable request for correspondence authors.

## Abbreviations

NIRF: Near-infrared fluorescence
DS: Dextran sulfate
Ce6: Chlorin e6
ASCVD: Atherosclerotic cardiovascular disease
^1^O_2_: Singlet oxygen
ROS: Reactive oxygen species
SMCs: Smooth muscle cells
ECs: Endothelial cells
SR-A: Scavenger receptor-A
LDL: Low-density lipoprotein
CDI: Carbonyldiimidazole
TEM: Transmission electron microscopy
UV/Vis: Ultraviolet/visible
SOSG: Singlet oxygen sensor green
IF: Immunofluorescence
LPS: Lipopolysaccharide
CCK-8: Cell counting kit-8
IVFM: Intravital fluorescence microscopy
pTBR: Plaque target-to-background ratio
CLSM: Confocal laser scanning microscopy
IHC: Immunohistochemistry
H&E: Hematoxylin and Eosin
PSR: Picrosirius Red
ORO: Oil red O
MerTK: Mer tyrosine kinase
BSA: Bovine serum albumin
OD: Optical density
FITC: Fluorescein isothiocyanate
SI: Signal intensity
TBST: Tris-buffered saline with Tween 20
SMF: Single-mode fiber
MMF: Multimode fiber
