# Supplementary material for: Targeted theranostic photoactivation on atherosclerosis
Source: J Nanobiotechnology. 2021 Oct 24;19:338. doi: 10.1186/s12951-021-01084-z (PMC8543964; doi:10.1186/s12951-021-01084-z)
Supplement: Supplementary file 1 — Additional file 1: Figure S1. FT-IR spectra of (1) DS and (2) DS-Ce6. Figure S2. a Representative confocal microscopic images and quantification of dose-dependent cellular uptake of DS-Ce6 in the activated macrophages. *P < 0.05, ***P < 0.001. b Comparison of the intracellular uptake of free Ce6 (5 μM) and DS-Ce6 (equiv. 5 μM Ce6) in the activated macrophages. To evaluate receptor-mediated endocytosis, the SR-A ligand DS was pre-treated for 1 h before DS-Ce6 incubation (DS + DS-Ce6). ***P < 0.001. Scale bar = 50 μm. c Comparison of the intracellular uptake of DS-Ce6 (equiv. 5 μM Ce6) in ECs, SMCs, and RAW264.7 macrophages treated with LPS or LPS with LDL. ***P < 0.001 compared to ECs, and ###P < 0.001 compared to SMCs. Scale bar = 30 μm. d Viability of activated macrophages treated with different concentrations of DS-Ce6, DS, and Ce6 with laser irradiation (670 nm, 50 mW). **P < 0.01. e DS-Ce6 photoactivation-induced apoptosis according to the time after laser irradiation. Annexin V (green) and DAPI (blue)-stained images of the activated macrophages that received DS-Ce6 treatment (equiv. 5 μM Ce6) and laser irradiation (670 nm, 50 mW). ***P < 0.001. Scale bar = 50 μm. f DS-Ce6 photoactivation-induced autophagy flux according to the time after laser irradiation. IF staining of LC3 (green), p62 (red), and DAPI (blue) in the activated macrophages that received DS-Ce6 treatment (equiv. 5 μM Ce6) and laser irradiation (670 nm, 50 mW). Scale bar = 25 μm. ***P < 0.001. Figure S3. a Quantification of whole-body fluorescence in the lateral and prone positions at 0 and 5 min and 1, 3, 6, 9, 12, 24, and 48 h post-injection (n = 3). b Quantification of Ce6 fluorescence intensity within plaques treated with DS-Ce6 or free Ce6. *P < 0.05. c Co-localization of DS-Ce6 (CLSM) and macrophages (Mac3) within the atheroma (red arrow). Scale bar = 100 μm. [file 12951_2021_1084_MOESM1_ESM.doc]

**Supplementary Information**

**Targeted theranostic photoactivation on atherosclerosis**

Joon Woo Song^1, †^, Jae Won Ahn^2, †^, Min Woo Lee^3^, Hyun Jung Kim^1^, Dong Oh Kang^1^, Ryeong Hyun Kim^1^, Un Gyo Kang^4^, Yeon Hoon Kim^4^, Jeongmoo Han^4^, Ye Hee Park^1^, Hyeong Soo Nam^4^, Hongki Yoo^4, *^, Kyeongsoon Park^2, *^, Jin Won Kim^1, *^

^1^ Multimodal Imaging and Theranostic Laboratory, Cardiovascular Center, Korea University Guro Hospital, Seoul, Republic of Korea

^2^ Department of Systems Biotechnology, Chung-Ang University, Anseong, Republic of Korea

^3^ Department of Biomedical Engineering, Hanyang University, Seoul, Republic of Korea

^4^ Department of Mechanical Engineering, KAIST, Daejeon, Republic of Korea

^†^ Joon Woo Song and Jae Won Ahn contributed equally to this work

**^*^Corresponding authors:**

Jin Won Kim, M.D., Ph.D., F.A.C.C.

Cardiovascular Center, Korea University Guro Hospital, 148, Gurodong-ro, Guro-gu, Seoul 08308, Republic of Korea, Tel.: 82-2-2626-3021, Fax: 82-2-863-1109, E-mail: [kjwmm@korea.ac.kr](mailto:kjwmm@korea.ac.kr)

Kyeongsoon Park, Ph.D.

Department of Systems Biotechnology, Chung-Ang University, Anseong, Gyeonggi-do 17546, Republic of Korea, Tel.: 82-31-670-3357, Fax: 82-31-675-1381, E-mail: [kspark1223@cau.ac.kr](mailto:kspark1223@cau.ac.kr)

Hongki Yoo, Ph.D.

Department of Mechanical Engineering, KAIST, 291 Daehak-ro, Yuseong-gu, Daejeon 34141, Republic of Korea, Tel.: 82-42-350-3243, E-mail: [h.yoo@kaist.ac.kr](mailto:h.yoo@kaist.ac.kr)

**Multichannel confocal laser scanning IVFM system**

In vivo optical imaging of carotid plaques and CLSM imaging of cryosections were conducted by multichannel laser confocal IVFM system as previously reported [1, 2]. Briefly, the IVFM instrument was adopted with upright epi-fluorescence configuration to increase the accessibility to murine carotid arteries. High-speed imaging acquisition up to 4 frames/s at 1024 × 1024 pixels was achieved using a resonant scanning mirror (Cambridge Technology, USA), and a large imaging field of view (2.4 mm × 2.4 mm) was acquired using a 10×/0.3-NA objective lens. FITC and Ce6 were excited using 488 nm and 633 nm laser, respectively. The fluorescent emissions signals were detected with a photomultiplier tube (Hamamatsu Photonics, Japan) through multiband dichroic bean splitter (Semrock, USA), multiband emission filter (Semrock), fiber-coupled beam combiner (SIFAM) and a variable confocal pinhole (Thorlabs, USA). The axial 400 μm range was scanned using a piezoelectric objective positioner (Piezosystem Jena GmbH, Germany), and carotid artery was imaged entirely by stacking multiple axially scanned images.

**Laser irradiation system**

A therapeutic laser irradiation system was developed for the stable and uniform light illumination of target lesions. A fiber-coupled laser diode (Blue Sky Research, Milpitas, CA, USA) with a wavelength of 658 nm was used as the photoactivation light source. Because the temperature of the laser diode was kept constant by a thermoelectric cooler, a stable laser output could be obtained. The fiber-coupled laser output was single-mode with a beam profile generally having a Gaussian distribution. To achieve a uniform beam distribution, the single-mode fiber (SMF) of the fiber-coupled laser diode was connected to a multimode fiber (MMF; Molex, Lisle, IL, USA) with a modifiable beam profile. To improve the uniformity of the MMF output, mode scrambling was applied using a mode scrambler consisting of a direct current motor and a twisted MMF. Uniformity-improved MMF output was focused using a plano-convex lens (THORLABS, Newton, NJ, USA).

**Additional file 1: Figures**


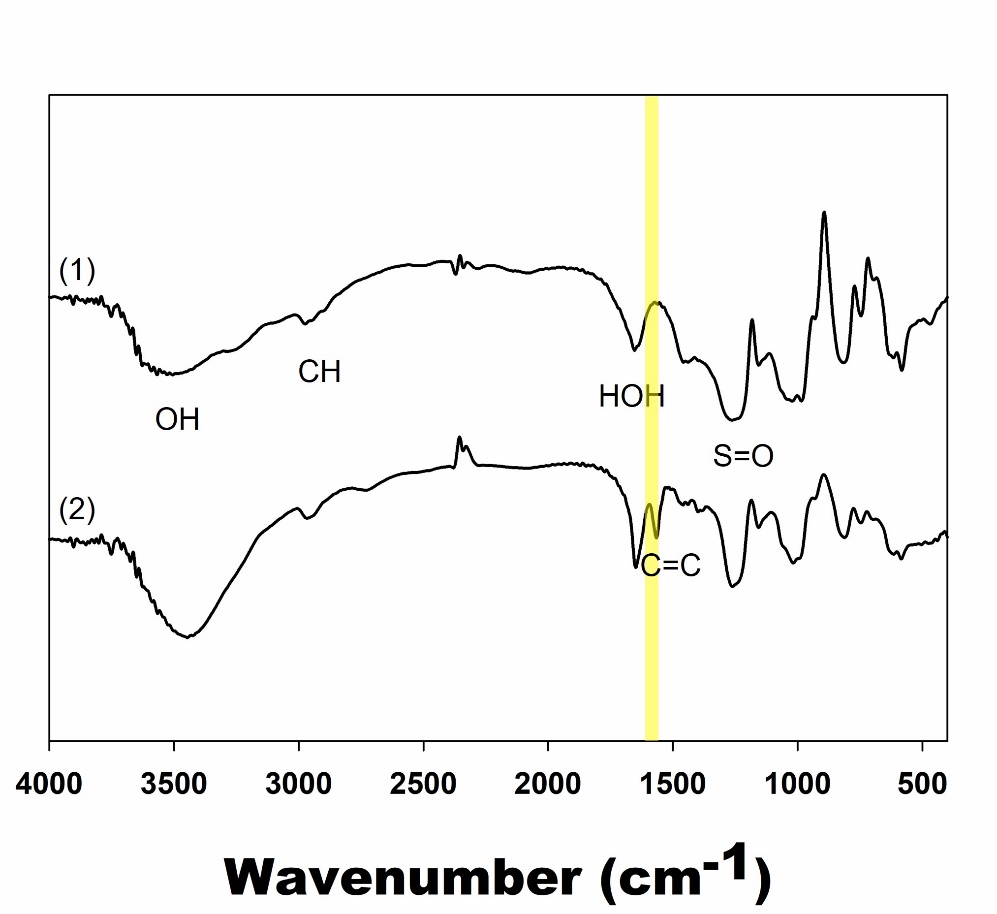


**Fig. S1** FT-IR spectra of (1) DS and (2) DS-Ce6.

**
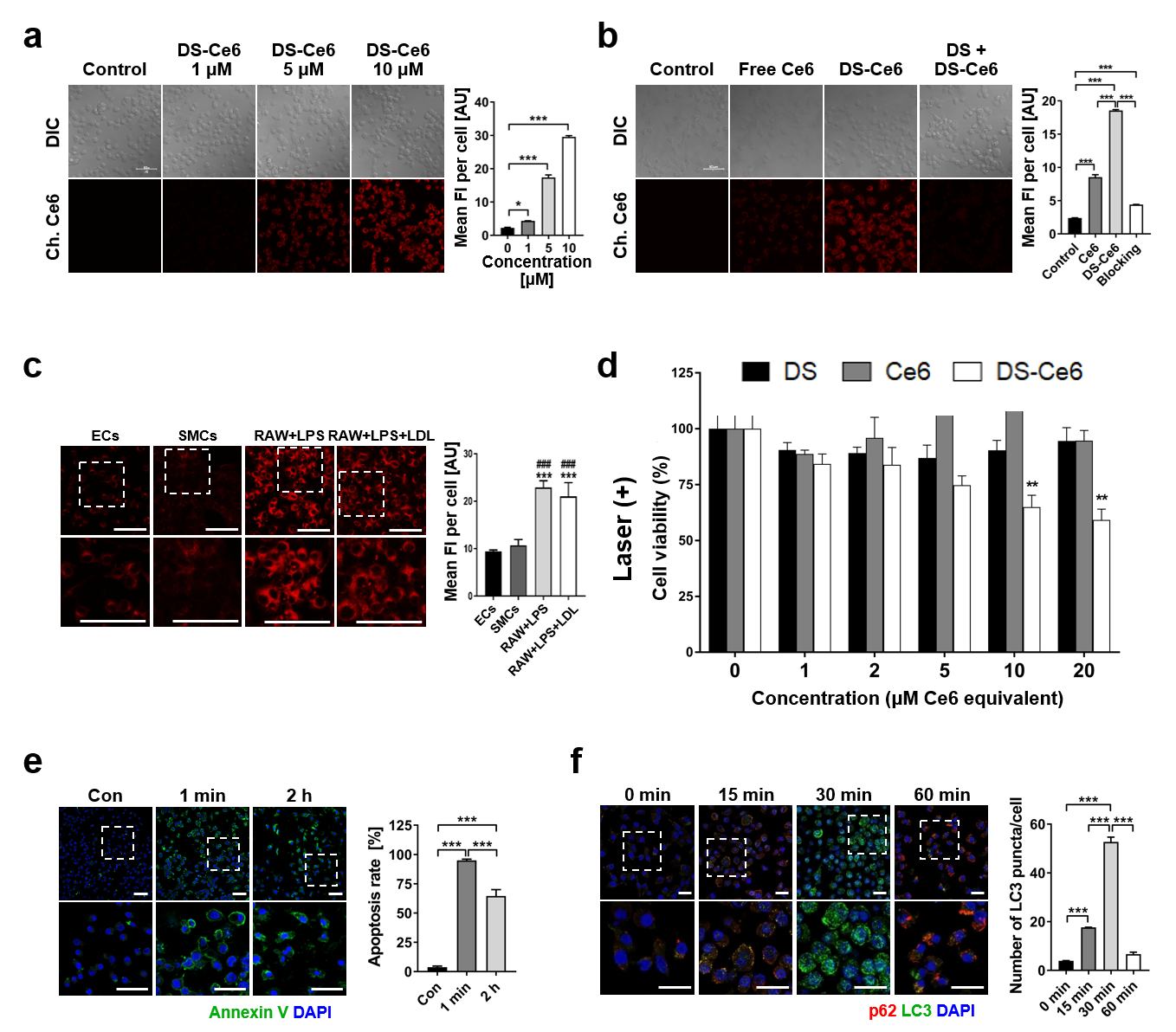
**

**Fig. S2 a** Representative confocal microscopic images and quantification of dose-dependent cellular uptake of DS-Ce6 in the activated macrophages. *^*^P* < 0.05, *^***^P* < 0.001. **b** Comparison of the intracellular uptake of free Ce6 (5 μM) and DS-Ce6 (equiv. 5 μM Ce6) in the activated macrophages. To evaluate receptor-mediated endocytosis, the SR-A ligand DS was pre-treated for 1 h before DS-Ce6 incubation (DS + DS-Ce6). *^***^P* < 0.001. Scale bar = 50 μm. **c** Comparison of the intracellular uptake of DS-Ce6 (equiv. 5 μM Ce6) in ECs, SMCs, and RAW264.7 macrophages treated with LPS or LPS with LDL. *^***^P* < 0.001 compared to ECs, and ^###^*P* < 0.001 compared to SMCs. Scale bar = 30 μm. **d** Viability of activated macrophages treated with different concentrations of DS-Ce6, DS, and Ce6 with laser irradiation (670 nm, 50 mW). ^**^*P* < 0.01. **e** DS-Ce6 photoactivation-induced apoptosis according to the time after laser irradiation. Annexin V (green) and DAPI (blue)-stained images of the activated macrophages that received DS-Ce6 treatment (equiv. 5 μM Ce6) and laser irradiation (670 nm, 50 mW). *^***^P* < 0.001. Scale bar = 50 μm. **f** DS-Ce6 photoactivation-induced autophagy flux according to the time after laser irradiation. IF staining of LC3 (green), p62 (red), and DAPI (blue) in the activated macrophages that received DS-Ce6 treatment (equiv. 5 μM Ce6) and laser irradiation (670 nm, 50 mW). Scale bar = 25 μm. *^***^P* < 0.001.

**
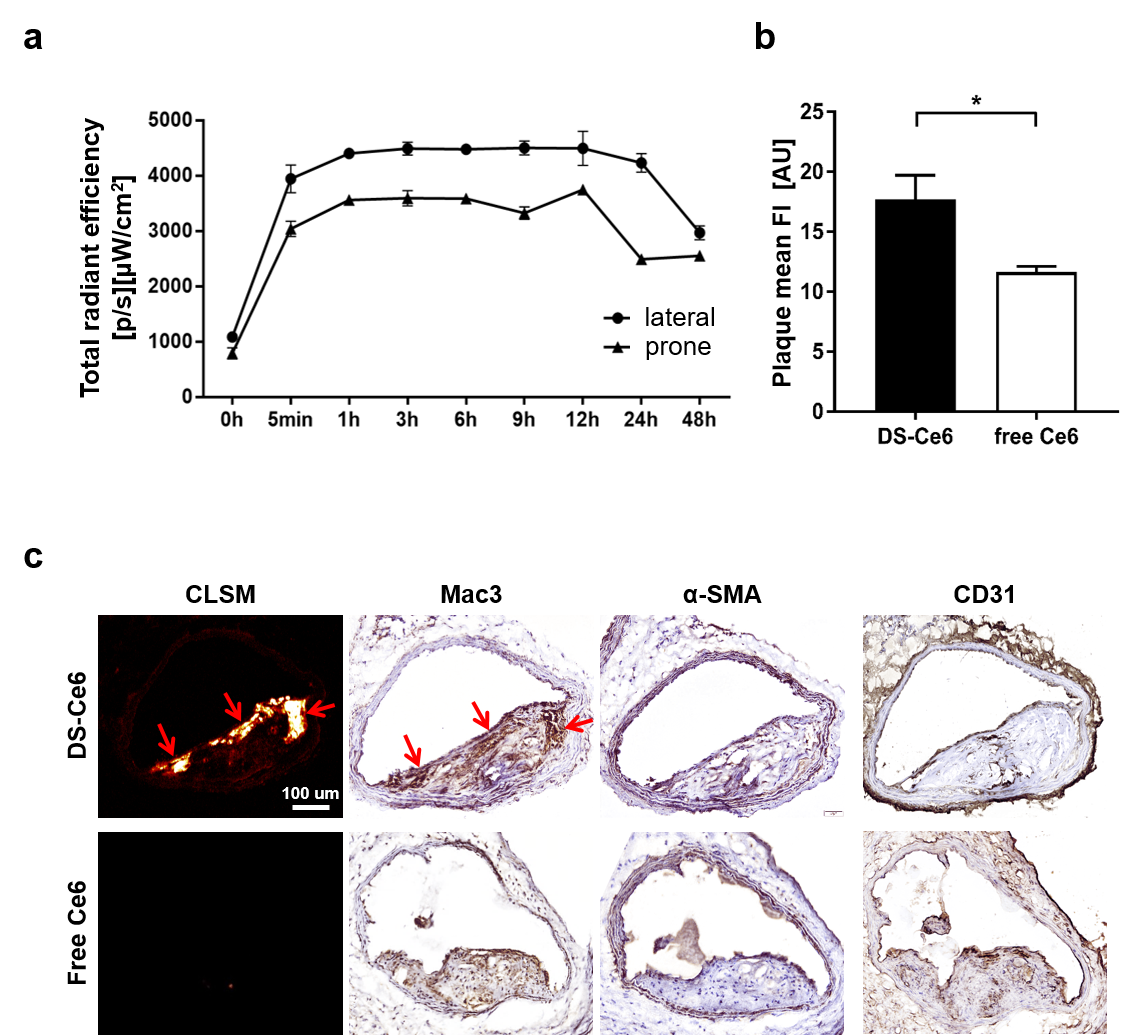
**

**Fig. S3 a** Quantification of whole-body fluorescence in the lateral and prone positions at 0 and 5 min and 1, 3, 6, 9, 12, 24, and 48 h post-injection (n = 3). **b** Quantification of Ce6 fluorescence intensity within plaques treated with DS-Ce6 or free Ce6. ^*^*P* < 0.05. **c** Co-localization of DS- Ce6 (CLSM) and macrophages (Mac3) within the atheroma (red arrow). Scale bar = 100 μm.

**References**

1. Choi JY, Ryu J, Kim HJ, Song JW, Jeon JH, Lee DH, Oh DJ, Gweon DG, Oh WY, Yoo H, et al: **Therapeutic Effects of Targeted PPAR Activation on Inflamed High-Risk Plaques Assessed by Serial Optical Imaging In Vivo.** *Theranostics* 2018, **8:**45-60.

2. Kim JB, Park K, Ryu J, Lee JJ, Lee MW, Cho HS, Nam HS, Park OK, Song JW, Kim TS, et al: **Intravascular optical imaging of high-risk plaques in vivo by targeting macrophage mannose receptors.** *Sci Rep* 2016, **6:**22608.
